# Multi‐omics integration reveals the oncogenic role of eccDNAs in diffuse large B‐cell lymphoma through STING signalling

**DOI:** 10.1002/ctm2.1815

**Published:** 2024-08-25

**Authors:** Zijuan Wu, Wei Zhang, Luqiao Wang, Jiayan Leng, Yongle Li, Zhou Fan, Mengtao Zhan, Lei Cao, Yongning Jiang, Yan Jiang, Bing Sun, Jianxin Fu, Jianyong Li, Wenyu Shi, Hui Jin

**Affiliations:** ^1^ Lymphoma Center, Department of Hematology, the First Affiliated Hospital of Nanjing Medical University, Jiangsu Province Hospital Nanjing Medical University Nanjing China; ^2^ Key Laboratory of Hematology, Nanjing Medical University Nanjing China; ^3^ Jiangsu Key Lab of Cancer Biomarkers, Prevention and Treatment, Collaborative Innovation Center for Personalized Cancer Medicine Nanjing Medical University Nanjing China; ^4^ Department of Hematology, Suqian Hospital Jiangsu Province Hospital Suqian China; ^5^ Department of Hematology, Sir Run Run Shaw Hospital Zhejiang University School of Medicine Hangzhou China; ^6^ Department of Hematology Affiliated People's Hospital of Jiangsu University Zhenjiang China; ^7^ Department of Oncology Affiliated Hospital of Nantong University Nantong China; ^8^ The Central Research Laboratory The First Affiliated Hospital of Soochow University Suzhou China

**Keywords:** Diffuse large B‐cell lymphoma, DNA damage, EccDNAs, STING signalling

## Abstract

**Background:**

Extrachromosomal circular DNAs (eccDNAs), a type of double‐stranded DNAs (dsDNAs) that facilitate the activation of the DNA sensing machinery, have been implicated in the progression and prognosis of various diseases. While the roles of eccDNAs remain contentious, their significance in diffuse large B‐cell lymphoma (DLBCL) has not been reported.

**Methods:**

Circular DNA sequencing (circle‐seq) was used to demonstrate the expression profile of eccDNAs in DLBCL, and atomic force microscopy to validate the presence of eccDNAs. CCK‐8 and scRNA‐seq techniques were employed to uncover the activation of eccDNA in the STING pathway, leading to enhanced cell proliferation. Chemotherapeutic drugs were used to test the hypothesis that DNA damage induces the production of eccDNA, thereby activating the STING pathway independent of cGAS. GEO databases were used for verification of the prognosis of the eccDNA‐related genes, and animal models were used to investigate the synergistic effects of DNA damage therapy in combination with STING inhibitors on anti‐tumour responses.

**Results:**

EccDNAs were widely expressed in DLBCL and associated with the prognosis of patients. Elevated abundance of eccDNAs promoted the progression of DLBCL. Chemotherapeutic drugs‐induced DNA damage triggered the generation of eccDNAs, resulting in the activation of the STING signalling in a cGAS‐independent manner. Moreover, inhibition of STING exerted a synergistic anti‐tumour effect with cisplatin.

**Conclusions:**

EccDNAs induced by DNA damage exert an oncogenic role in DLBCL via activating the STING signalling independently of cGAS. This finding offers a rational therapeutic strategy combining chemotherapy with targeting STING.

**Highlights:**

- EccDNAs induced by DNA damage exert an oncogenic role in DLBCL via activating the STING signalling independently of cGAS.
- The combined treatment of chemotherapeutic drugs with STING inhibitor significantly delayed the tumor progression, providing new insights into the therapeutic strategy for patients with DLBCL, particularly the relapsed and/or refractory (R/R) ones.

## Graphical abstract

1. Multi‐omics analysis uncovers the expression profile and oncogenic role of eccDNAs in DLBCL.
2. EccDNAs induce the activation of the STING pathway independently of cGAS, promoting the proliferation of DLBCL cells.
3. Chemotherapeutic drugs‐induced DNA damage triggers the generation of eccDNAs, and the combined treatment of chemotherapeutic drugs with STING inhibitor significantly effectively prevents the progression of DLBCL.

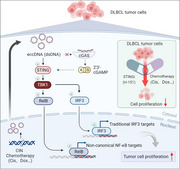

## 1 INTRODUCTION

Diffuse large B‐cell lymphoma (DLBCL) is the most predominant form of non‐Hodgkin lymphoma worldwide, with an annual occurrence of 150 000 new cases.^1^ While the majority of patients can achieve remission through the administration of R‐CHOP (rituximab, cyclophosphamide, doxorubicin, vincristine, and prednisone) immunochemotherapy, a significant proportion (30–40%) still experience treatment failure and ultimately succumb to the disease.^2^, ^3^ While there has been some advancement in the fundamental research of DLBCL,^4^, ^5^ it is imperative to enhance efforts in deepening the comprehension of DLBCL pathogenesis and devising strategies to enhance patient prognosis.

Extrachromosomal circular DNAs (eccDNAs) are one of the types of double‐stranded DNAs (dsDNAs) that are present in a circular form. Derived from genomic DNA, eccDNAs operate independently of chromosomes.^6^ Genes such as MYC and EGFR encoded by eccDNAs were reported to amplify more effectively than chromosomal genes through the process of self‐replication.^7^, ^8^ The uneven segregation of eccDNAs may help to understand this phenomenon and offer novel insights into how eccDNAs contribute to oncogenesis.^9^ The formation of eccDNAs can be influenced by DNA damage repair pathways, chromothripsis, apoptosis, and other unknown factors.^10^ Chromothripsis results in genome rearrangement within these biological processes and the formed sequences can be linked and cyclized to form eccDNAs, increasing the copy number and expression of oncogenes.^11^ Potential applications of eccDNAs in cancer diagnosis, targeted therapy, and prognostic assessment have been explored.^12^ However, the role of eccDNAs in DLBCL remains unclear.

cGAS‐STING signalling consists of the synthase for the second messenger cyclic GMP‐AMP (cGAS) and the cyclic GMP‐AMP receptor stimulator of interferon genes (STING), which catches abnormal DNAs (dsDNAs, microbial DNAs, cytosolic DNAs, released mitochondrial DNAs, etc.) to activate an innate immune response against viral infections.^13^, ^14^ Multifaceted roles of cGAS‐STING signalling in the regulation of autophagy, protein translation, metabolic homeostasis, DNA damage repair, cell senescence, cell death, etc., are demonstrated.^14^ Imbalanced cGAS‐STING signalling has been found to have involvement in malignant diseases and exhibited dual roles in tumours.^15^, ^16^, ^17^ For example, nuclear cGAS suppresses DNA repair and promotes tumour growth.^18^ Activation of the STING pathway and downstream noncanonical NF‐κB signalling drives the evolution of the tumour.^19^ Meanwhile, Hu et al.^20^ found that intrinsic STING activation in cancer cells helps to inhibit lung adenocarcinoma metastasis. The development of selective small‐molecule inhibitors and agonists targeting the cGAS‐STING axis has gained surprising efficacy in multiple diseases. Insights into the roles of the cGAS‐STING pathway are urgently needed to broaden our understanding of DLBCL.

In this study, for the first time, we used multiple omics sequencing and validation experiments to delineate the panorama of eccDNAs in DLBCL. The abundance of eccDNAs was found to be upregulated in both chemotherapy‐treated cells and DLBCL patients. Furthermore, we observed that DNA‐damaging chemotherapeutic agents triggered the generation of eccDNAs, which subsequently activated the STING pathway. Notably, the simultaneous administration of chemotherapeutic agents and STING inhibitors exhibited synergistic effects on cell and tumour growth. These findings emphasize the roles and underlying mechanisms of eccDNAs in DLBCL and present a promising therapeutic strategy that targets the STING pathway.

## 2 MATERIALS AND METHODS

### 2.1 Cell lines

Human DLBCL cell lines, including U2932, HBL1, FARAGE, CTB‐1, KIS1, BJAB, RIVA, SU‐DHL‐2, SU‐DHL‐4, SU‐DHL‐10, SU‐DHL‐6, DB, OCI‐LY19, WSU‐DLCL2, and DOHH2 were cultured in RPMI‐1640 medium supplemented with 10% fetal bovine serum (FBS) (Biochannel) and 1% penicillin/streptomycin (PS) (Gibco). SU‐DHL‐8 cell line was grown in RPMI‐1640 medium supplemented with 20% FBS and 1% PS, while OCI‐LY3 and MEDB1 cell lines were maintained in a complete IMDM medium (Gibco). The mouse B‐cell lymphoma cell line A20 grew in RPMI‐1640 medium supplemented with 10% FBS, 1% PS, and 0.05 mM 2‐mercaptoethanol.

### 2.2 Xenograft study

To establish the immune‐deficient and competent mice models, U2932 and A20 cells (1 × 10^7^ cells per animal) were subcutaneously injected into the armpit of the forelimb of 8‐week‐old NOD‐SCID and 5‐week‐old BALB/C mice, respectively. Cells were suspended in PBS and mixed with Matrigel at a 1:1 ratio. Mice were randomly divided into different groups and treated intraperitoneally every 2 days with H‐151 (4.2 mg/kg)/C‐176 (0.8 mg/kg), cisplatin (5 mg/kg), or a combination of H‐151/C‐176 and cisplatin. For in vivo injection, H‐151 and C‐176 were diluted in a salt solution containing 5% DMSO, 40% PEG, and 5% TWEEN, while cisplatin was diluted in a salt solution containing 20% DMSO, 40% PEG, and 5% TWEEN. Tumours and body weights were measured biweekly or triweekly. Tumour tissues were collected for further analysis when the mice were euthanized.

### 2.3 Statistical analysis

Data were analyzed using GraphPad Prism 8.0 software, and the results were represented as mean ± SD of independent biological replicates. Statistical analysis was performed as described in the figure legends. For overall survival (OS) analysis, bulk RNA‐seq transcriptome data and clinic information were obtained from three published datasets (GSE10846, GSE87371, and GSE31312). To assess the relationship between the target gene sets and clinical factors, ssGSEA was used to calculate a score for each gene set in the samples. The samples were then divided into high group (H group) and low group (L group) based on the optimal cut‐off value. Kaplan–Meier curves were used to compare the differences in OS and progression‐free interval between H and L groups, and *p*‐values were calculated using the log‐rank test with the Survival package in R. *p*‐values < 0.05 were considered statistically significant.

## 3 RESULTS

### 3.1 Expression patterns and characteristics of eccDNAs in DLBCL

**FIGURE 1:**
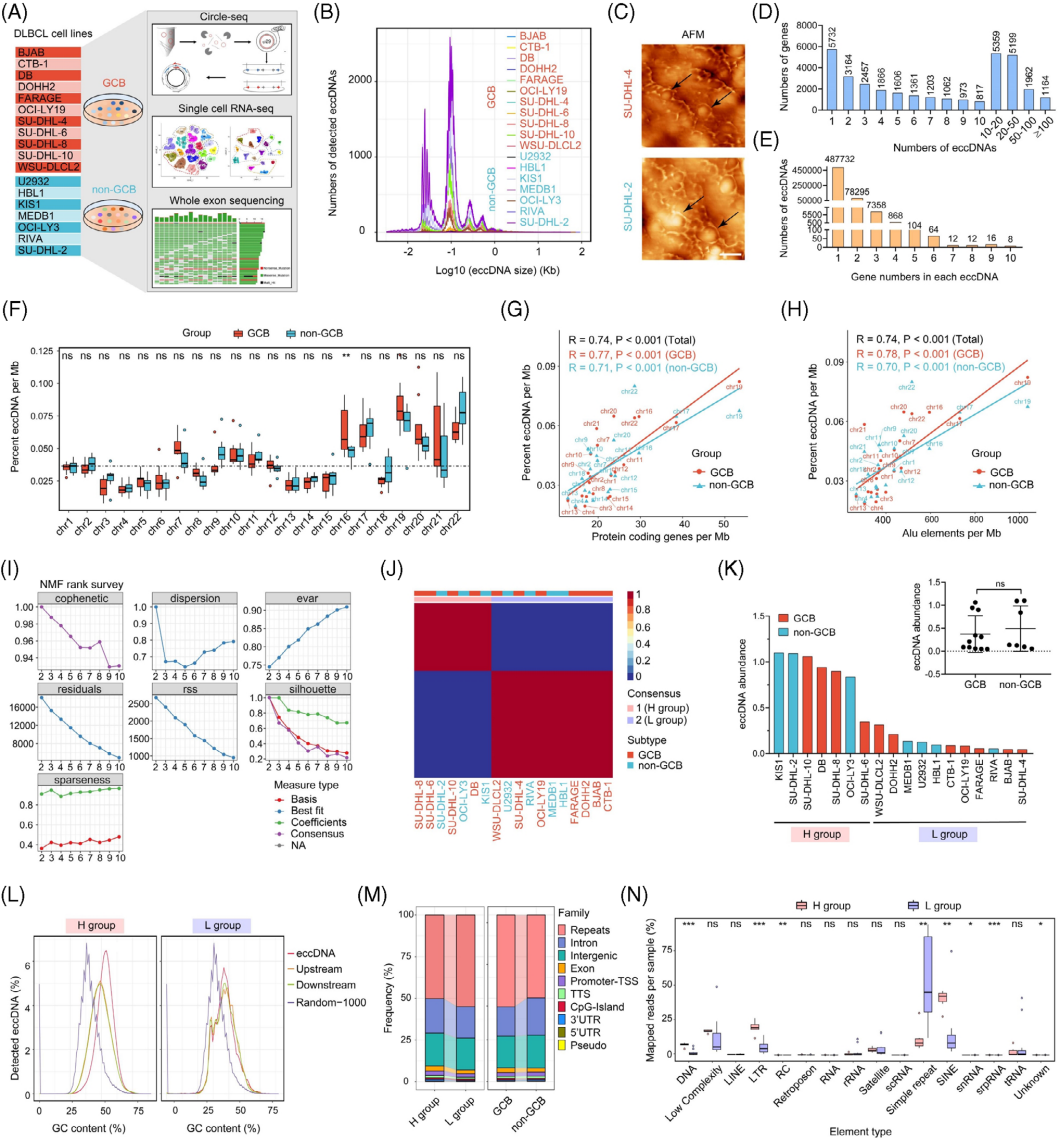
Identification and characterization of eccDNAs in 18 DLBCL cell lines. (A) Multi‐omics sequencing of 18 DLBCL cell lines. (B) The size distribution of eccDNAs in DLBCL cells. (C) AFM images of extracted eccDNAs in DLBCL cell lines. Scale bar, 200 nm. All imaging experiments were repeated at least three times. (D) Number of genes derived from different types of eccDNAs. (E) Number of eccDNA types amplifying 1 to 10 different genes. (F) Percentage of eccDNAs per Mb from different chromosomes in GCB and non‐GCB DLBCL groups. (G, H) Positive correlation between percentage of eccDNAs per Mb against protein‐coding genes per Mb and Alu elements per Mb in GCB and non‐GCB group cells. (I, J) Nonnegative matrix factorization (NMF) divides the DLBCL cell lines into H and L groups. (K) Abundance of eccDNAs in in GCB and non‐GCB DLBCL groups. (L) GC contents of eccDNA locus and regions immediately upstream and downstream from the eccDNAs compared with the genomic average in H/L groups, and GCB/non‐GCB groups. upstream, upper panel; downstream, lower panel; Orange, 1000 stretches upstream eccDNA locus (from eccDNA_start‐1000 to eccDNA_start); Red, eccDNA (from eccDNA_start to eccDNA_end); Green, 1000 stretches downstream eccDNA locus (from eccDNA_end to eccDNA_end+1000); Purple, 1000 random stretches of the genome of equivalent length as the eccDNA. (M) Frequency of eccDNAs in different classes of genomic regions from H/L groups, and GCB/non‐GCB groups. (N) Normalized mapping ratio of eccDNA reads in different types of elements from H and L groups.

To investigate the presence and expression patterns of eccDNAs in DLBCL, we conducted a multi‐omics study incorporating circle DNA sequencing (circle‐seq), whole exome sequencing (WES), and single‐cell RNA sequencing (scRNA‐seq) using 11 germinal centre B cell (GCB) and seven non‐GCB DLBCL cell lines (Figure 1A). EccDNAs were identified using the Circle‐Map software (Table S1). Notably, a strong positive correlation was observed between the number and abundance of eccDNAs (Figure S1A). Given that DLBCL cells are characterized by chromosomal instability (CIN) (Figure S2, S3, Table S1), we employed the weighted genome instability index (wGII) from our WES data as a representation of CIN. Our findings revealed that cells with elevated wGII levels exhibited higher levels of eccDNA abundance (Figure S1B). Analysis of the size distribution showed that eccDNAs measuring less than 100 bp constituted the primary subtype in DLBCL cells (Figure 1B). There was no difference in the average size of the eccDNAs between GCB and non‐GCB groups. Nevertheless, the peak values were higher in the non‐GCB group (Figure S1C). To validate the existence of eccDNAs, atomic force microscopy (AFM) was used to visualize the circular structure of eccDNAs (Figure 1C). Additionally, we isolated total DNAs from DLBCL cell lines and quantified the abundance of eccDNAs by enzymatic digestion followed by agarose gel electrophoresis (AGE). The results were aligned with those obtained from circle‐seq (Figure S1D). EccDNAs were found to map to either part or complete exons of protein‐coding genes. Astonishingly, 1164 genes engendered more than 100 distinct eccDNA species (Figure 1D), and more than 80 000 eccDNAs were derived from diverse genes (Figure 1E). The percentage of eccDNAs per megabase (Mb) from different chromosomes differed, but there was no difference in the proportion between the GCB and non‐GCB groups except for chr16 (Figure 1F). Furthermore, we observed a positive correlation between eccDNA frequency and the number of protein‐coding genes per Mb, suggesting a potential association between gene transcriptional activity and eccDNA generation (Figure 1G). We further analyzed the relationship between eccDNA frequency and the standardized amount of Alu element per Mb and found a positive correlation (Figure 1H). Subsequently, using non‐negative matrix factorization (NMF), we classified the 18 DLBCL cell lines into two groups based on high or low eccDNA abundance, referred to as the High group (H group) and the Low group (L group), respectively (Figure 1I, J). The abundance and numbers of eccDNAs exhibited a remarkable disparity between the H group and L group, with no discernible distinction observed between the GCB and non‐GCB groups (Figure 1K, Figure S1E). The heatmap showed the differentially expressed eccDNAs between the H group and L groups (Figure S1F). Remarkably, the enrichment of GC contents in eccDNA sequences was found in the H/L and GCB/non‐GCB groups compared with other genomic regions (Figure 1L, Figure S1G). We also explored the likely origins of eccDNAs by mapping them to various genomic regions and element types in the H/L and GCB/non‐GCB groups (Figure 1M,N, Figure S1H). Intriguingly, repeated sequences, which account for approximately 52.5% of the human genome, seemed to be more predisposed to form eccDNAs. Moreover, we observed a conspicuous enrichment of eccDNAs within the simple repeat and short interspersed nuclear element regions. Notably, the disparity between the H and L groups was particularly striking.

### 3.2 ScRNA‐seq analysis demonstrates the potential roles of eccDNAs in DLBCL

**FIGURE 2:**
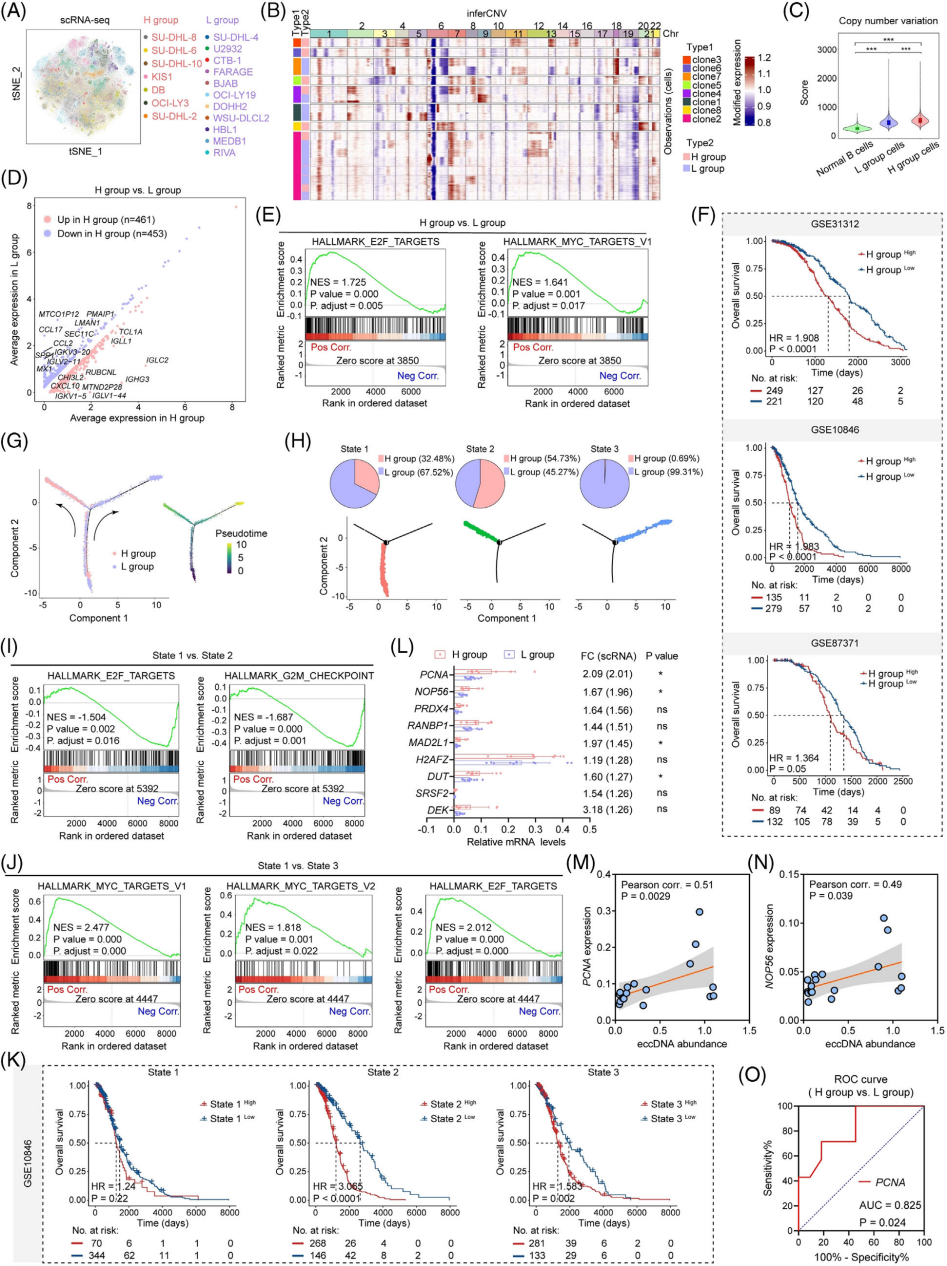
The results of scRNA‐seq analysis demonstrated the potential roles of eccDNAs in DLBCL. (A) t‐SNE plot of DLBCL cells derived from 18 cell lines. (B) Representative inferred copy number variation of the H and L group cells based on scRNA‐seq data. (C) The copy number variation score of normal B cells and DLBCL cells from different groups. (D) The heatmap shows the differentially expressed genes in H and L groups. (E) GSEA shows the enriched pathways in H group compared with L group. NES, the normalized enrichment score. (F) Survival analysis of DLBCL patients with high or low expression of genes that are highly expressed in the H group from three collected independent cohorts. (G) Single‐cell trajectory analysis of H and L group cells. (H) States of cells in different developmental trajectories based on scRNA‐seq data. (I, J) GSEA shows the enriched pathways in state 1 compared with state 2 and 3. (K) Survival analysis of relatively highly expressed genes in different states. (L) The expression of 9 overlapped genes that are upregulated in H groups detected by scRNA‐seq as well as involved in the pathways of E2F targets and MYC targets detected by qRT‐PCR (*n* = 18). (M, N) Correlation between the relative levels of PCNA, NOP56, and eccDNA abundance in DLBCL cell lines (*n* = 18). (O) The receiver operating characteristic (ROC) curve of PCNA is built to differentiate H and L groups. All results are expressed as the mean ± SD of three independent experiments. *p‐*values were calculated by two‐way ANOVA (C, L) and log rank test (F, K). ns, not significant; *p < 0.05, **p < 0.01, ***p < 0.001.

To delve into the potential roles of eccDNAs in DLBCL, we employed scRNA‐seq in 18 DLBCL cell lines and obtained 142 586 high‐quality cells from 184 652 cells after quality filtering. T‐distributed stochastic neighbour embedding (t‐SNE) visualization of filtered cells from H and L groups was shown (Figure 2A). InferCNV analysis revealed a higher copy number variation score in DLBCL cells, particularly in the H group cells, compared with normal B cells from our healthy volunteers (Figure 2B,C). Furthermore, through differential analysis, we identified 461 upregulated and 453 downregulated genes with a fold change of more than 1.2 in H group cells (Figure 2D). Gene set enrichment analysis (GSEA) analysis demonstrated that upregulated genes in the H group were significantly enriched in pathways closely associated with cell proliferation, such as E2F targets and MYC targets (Figure 2E). Additionally, our study revealed the different transcription factors (TFs) in the cells from H and L groups (Figure S4A). Notably, we observed the presence of *FOS*, *JUN*, and *E2F1*, implicated in cell proliferation, in the H group cells (Figure S4B). Three GEO datasets (GSE31312, GSE10846, and GSE87371) were used to validate the role of eccDNAs in DLBCL prognosis. Utilizing the top highly expressed 100 genes in the H group compared with the L group as a panel, we performed an overall survival (OS) analysis. The results revealed that patients with elevated levels of these genes experienced significantly worse outcomes (Figure 2F). Subsequently, to elucidate the trajectory of differentiation of DLBCL cells, we performed a pseudo‐time analysis based on B‐cell‐differentiation‐related genes. The results indicated that cells in the initial stage of differentiation (state 1) developed into two distinct differentiation directions, characterized by either high (state 2) or low eccDNA abundance (state 3) (Figure 2G,H). By conducting GSEA on cells from the three states, we observed significant enrichment of pathways involved in cell proliferation in state 2 (predominantly consisting of H group cells) (Figure 2H,I). In contrast, the pathways were more enriched in state 1 than in state 3 (predominantly consisting of L group cells) (Figure 2H,J). Furthermore, by comparing the differently expressed genes in the three states, we constructed the panels of the top 100 genes that were upregulated in each state. OS analysis showed patients with highly expressed genes in state 2 compared with other states had the highest hazard ratio (Figure 2K). To ascertain if the elevated levels of eccDNAs impact the proliferative capacity of cells, we performed qRT‐PCR assays on nine genes that were upregulated in the H group and were implicated in the MYC and E2F target pathways (Figure 2L). Among these genes, we verified the upregulation of *PCNA*, *NOP56*, *MAD2L1*, and *DUT* in the H group. Interestingly, the expression levels of *PCNA* and *NOP56* were positively correlated with eccDNA abundance (Figure 2 M,N, Figure S5A). Receiver‐operating characteristic curves and the corresponding area under the curve were generated, yielding values of 0.825 and 0.779 for *PCNA* and *NOP56*, respectively (Figure 2O, Figure S5B).

### 3.3 EccDNAs promote cell proliferation through activating STING signalling independently of cGAS

**FIGURE 3:**
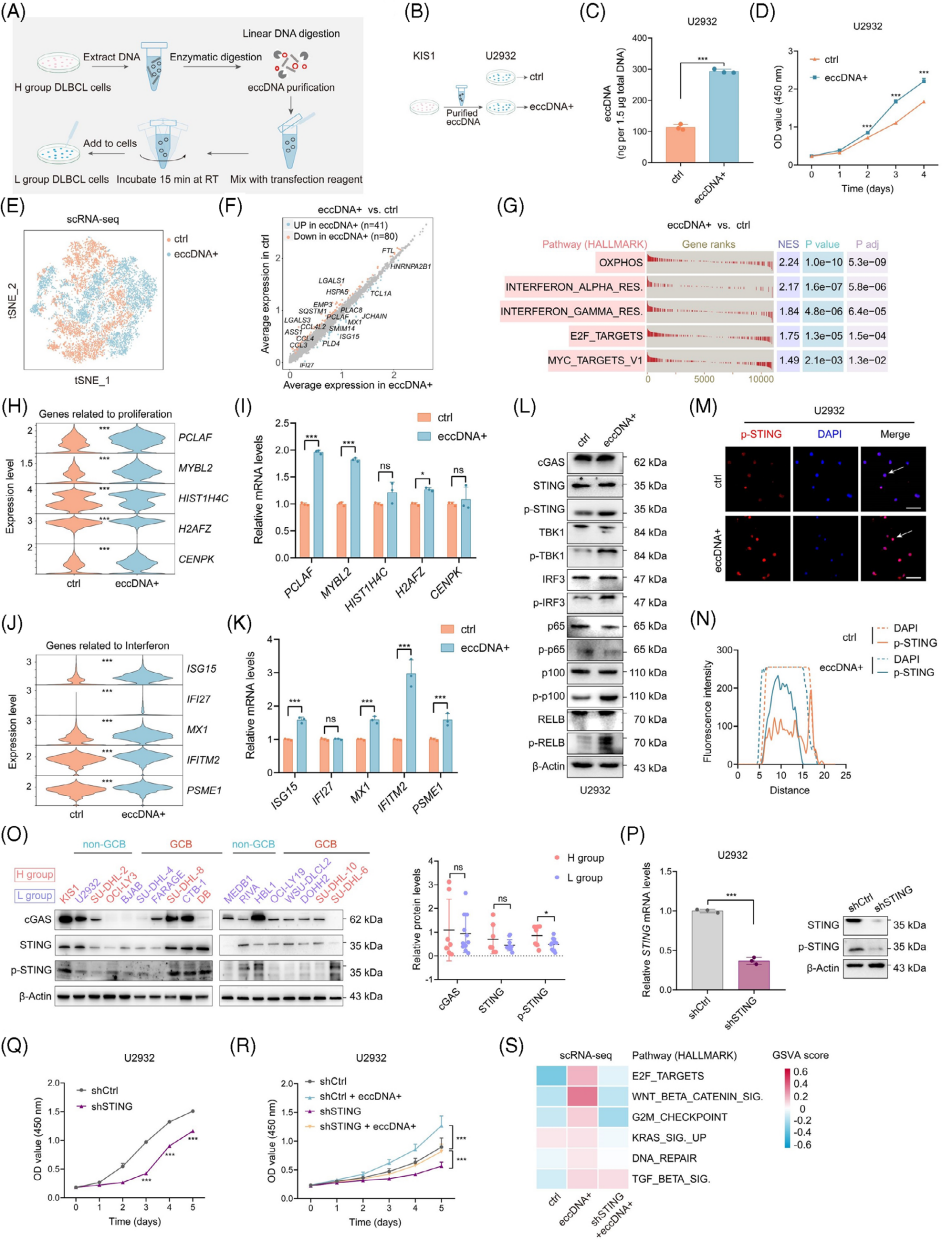
EccDNAs promote cell proliferation by activating the STING pathway. (A) The workflow of eccDNA extraction, purification, and transfection. (B) EccDNAs extracted from the KIS1 cell line in H group are transfected into L group cells U2932. (C) The quantified abundance of eccDNAs in the eccDNA+ (cells with eccDNA transfection) and ctrl groups (cells without eccDNA transfection). (D) Analysis of the proliferative ability of cells from eccDNA+ and ctrl groups. (E) tSNE visualization of cells with or without eccDNA transfection based on scRNA‐seq data. (F) Differentially expressed genes in cells with or without eccDNA transfection. (G) GSEA shows the enriched pathways in eccDNA+ cells compared with ctrl cells. (H, I) The top 5 upregulated genes related to proliferation in the eccDNA+ group were observed by scRNA‐seq (H), and validated by qRT‐PCR analysis (I). (J, K) The top five upregulated genes related to interferon in eccDNA+ group observed by scRNA‐seq (J), and validated by qRT‐PCR analysis (K). (L) The expression of proteins in the cGAS‐STING pathway in U2932 cells with or without eccDNA transfection. (M, N) IF assay shows the levels of p‐STING in eccDNA+ and ctrl cells. Scale bar = 50 µm. (O) The expression of cGAS, STING, and p‐STING qualified by western blotting (WB) and compared between H and L groups. (P) Relative levels of STING after STING knockdown in U2932 cells. (Q) Proliferation ability of cells with different levels of STING in U2932 cells. (R) Growth curves of U2932 cells with STING silencing and/or eccDNA transfection. (S) Gene set variation analysis (GSVA) shows the enriched pathways in cells with STING silencing and/or eccDNA transfection. All results are expressed as the mean ± SD of three independent experiments. *p*‐values were calculated by two‐tailed Student's *t*‐test (C, D, P, Q), and two‐way ANOVA (H–K, O, R). ns, not significant; *p < 0.05, **p < 0.01, ***p < 0.001.

To validate the impact of eccDNAs on cell proliferation, we transfected eccDNAs that were extracted from H group cells into L group cells (Figure 3A). Increased eccDNA abundance was observed after transfection (Figure 3B,C, Figure S6A,B). As expected, cells transfected with eccDNAs (referred to as eccDNA+ cells) showed a significantly higher propagation rate than control cells (referred to as ctrl cells) (Figure 3D, Figure S6C). ScRNA‐seq was performed and t‐SNE visualization of filtered cells from eccDNA+ and ctrl groups was shown (Figure 3E). Through differential analysis, we identified 41 upregulated and 80 downregulated genes in eccDNA+ group cells (Figure 3F). GSEA revealed that eccDNA+ cells were enriched in pathways related to proliferation and interferon response (Figure 3G). Next, a panel of the top 100 highly expressed genes in the eccDNA+ group compared with the ctrl group was selected for OS analysis. Worse outcomes were observed in patients with high expression levels of these genes in GSE10846 and GSE31312 datasets (Figure S6D). The cells were then partitioned into 16 clusters (Figure S6E). Clusters 1, 7, and 11, which were mainly from eccDNA+ cells, exhibited a greater enrichment in interferon response and proliferation‐related pathways than clusters 3, 4, 8, 14, and 15 that from ctrl cells (Figure S6F,G). To confirm these findings, we performed qRT‐PCR assays and ensured that the top five upregulated genes acquired from the scRNA‐seq data and involved in cell proliferation and interferon response were highly expressed in eccDNA+ cells (Figure 3H–K). Given that cells were enriched in immune response pathways after the transfection of eccDNAs, and the cGAS‐STING pathway has been identified as a crucial DNA sensor activated by cytosolic dsDNAs and involved in the inflammatory response, we proceeded to investigate whether eccDNAs exerted function through the cGAS‐STING pathway.^16^ Phosphorylated STING (p‐STING) and its downstream proteins p‐TBK1, p‐IRF3, and noncanonical NF‐κB signalling (p‐RELB) exhibited heightened levels after the transfection with eccDNAs (Figure 3L, Figure S6H). IF assay confirmed the enhanced expression of p‐STING again (Figure 3M,N, Figure S6I). Furthermore, the expression of p‐STING was significantly higher in the H group compared with the L group, while cGAS expression showed no difference (Figure 3O). Next, we knocked down *cGAS* and *STING*, respectively, and found that the inhibition of *STING*, rather than *cGAS*, suppressed the proliferation of DLBCL cells (Figure 3P,Q, Figure S6J–O). However, the inhibitory effect was partially counteracted by enhanced abundance of eccDNAs (Figure 3L, Figure S6P). Consistent results were observed via gene set variation analysis using scRNA‐seq data, further supporting the notion that eccDNAs promote cell proliferation in DLBCL tumour cells (Figure 3S).

### 3.4 Chemotherapeutic drugs‐induced DNA damage elicits the generation of eccDNAs and activates the STING signalling

**FIGURE 4:**
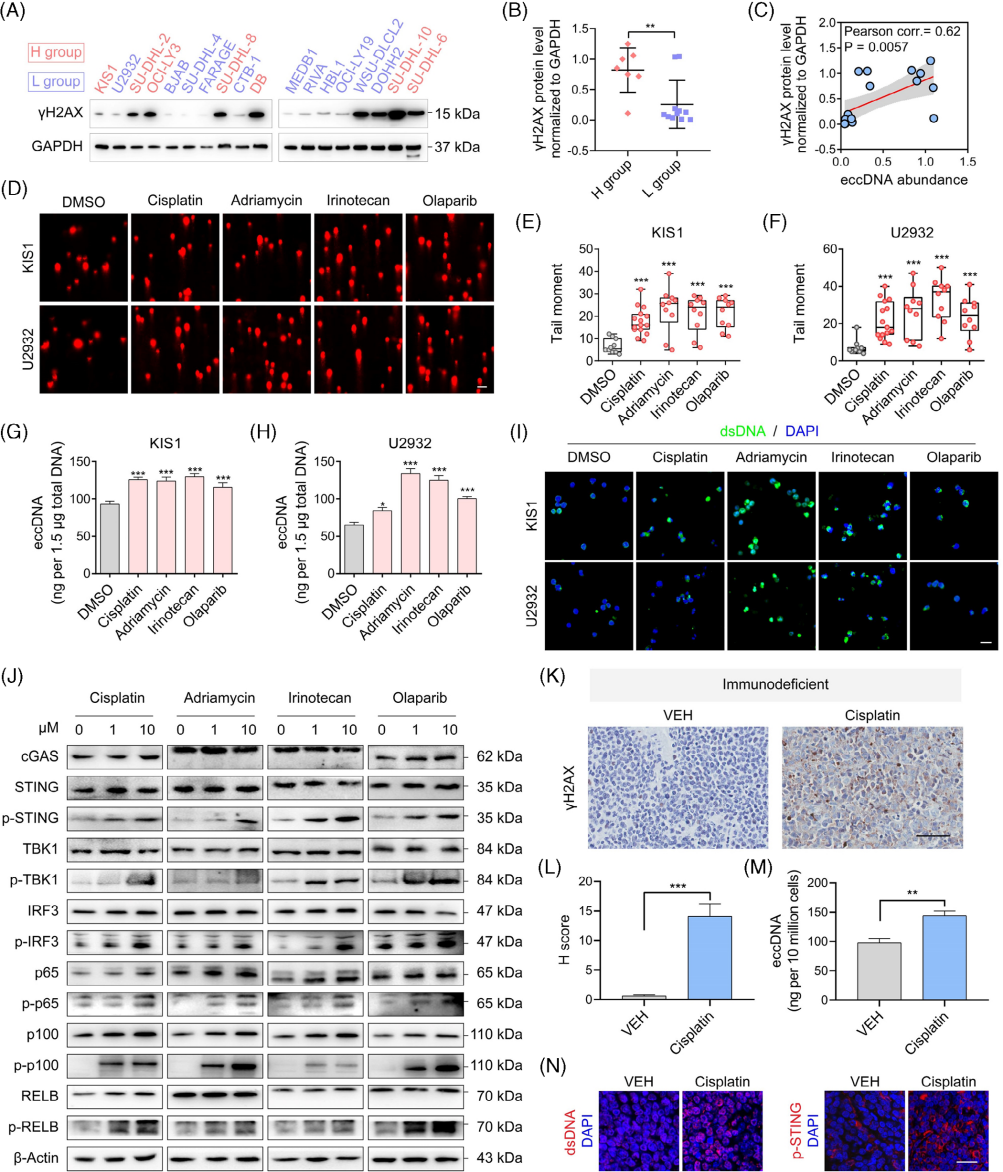
DNA‐damaging chemotherapeutic agents promote the generation of eccDNAs and activation of STING signalling. (A) WB detects the expression of γH2AX, a marker of DNA double‐strand breaks, in 18 DLBCL cell lines. (B) The quantification and comparison of the levels of γH2AX in H and L group cells. (C) Correlation between γH2AX protein levels and eccDNA abundance in different DLBCL cell lines. (D) Representative images of DLBCL cells treated with DNA‐damaging agents (5 µM) in the comet assay. Scale bar = 50 µm. (E, F) Quantification of tail‐moment in the comet assays. *n* = 10 cells per cell line and condition. (G, H) Quantification of eccDNA abundance of cells treated with DNA‐damaging agents (1 µM). (I) Representative IF assay for dsDNA. Scale bar = 50 µm. (J) Western blot analysis of the levels of STING pathway in DLBCL cells treated with DNA‐damaging agents. Representative images of three independent experiments. (K–N) Representative images of immunohistochemistry (IHC) staining for γH2AX, scale bar, 50 µm, (K), IHC score of γH2AX (L), eccDNA abundance (M), and IF staining for dsDNA and p‐STING (N) in tumours from immunodeficient mice treated with vehicle (VEH) or cisplatin, scale bar, 20 µm. All results are expressed as the mean ± SD of three independent experiments. *p*‐values were calculated by two‐tailed Student's *t*‐test (B, E–H, L, M). *p < 0.05, **p < 0.01, ***p < 0.001.

Previous studies have reported that DNA damage could induce the production of eccDNAs.^21^ We detected the expression levels of phosphorylation of histone H2AX at Ser 139 (γH2AX), a marker for DNA damage, in DLBCL cell lines (Figure 4A). H group cells showed higher expression of γH2AX than the L group (Figure 4B). As anticipated, the expression of γH2AX and eccDNA abundance were positively correlated (Figure 4C). Moreover, the treatments with chemotherapeutic drugs such as cisplatin, doxorubicin, irinotecan, and targeted drug olaparib indeed induced DNA damage (Figure 4D–F). Besides, these drugs triggered the generation of eccDNAs (Figure 4G,H). The levels of dsDNAs were also found to escalate with DNA damage (Figure 4I). Concurrently, the expression of p‐STING, its downstream proteins, and proteins involved in non‐canonical NF‐κB signalling demonstrated significant enhancement (Figure 4J). To further validate the association between DNA damage and eccDNAs in vivo, we employed cisplatin treatment on immunodeficient mouse models established using U2932 cells. Immunohistochemical (IHC) assays confirmed the increased levels of γH2AX in tumours from cisplatin‐treated mice (Figure 4K,L). Similarly, the upregulation of eccDNAs was observed as well (Figure 4M). We also assessed the expression of dsDNA and p‐STING by IF assay and confirmed their increased levels in the cisplatin‐treated group (Figure 4N). To investigate whether these findings could be applied to mice with a normal function of the immune system, we established immunocompetent mouse models using the mouse B‐cell lymphoma cell line (A20) and observed consistent results (Figure S7A–D).

### 3.5 Inhibition of STING exerts synergistic anti‐tumour effect with cisplatin

**FIGURE 5:**
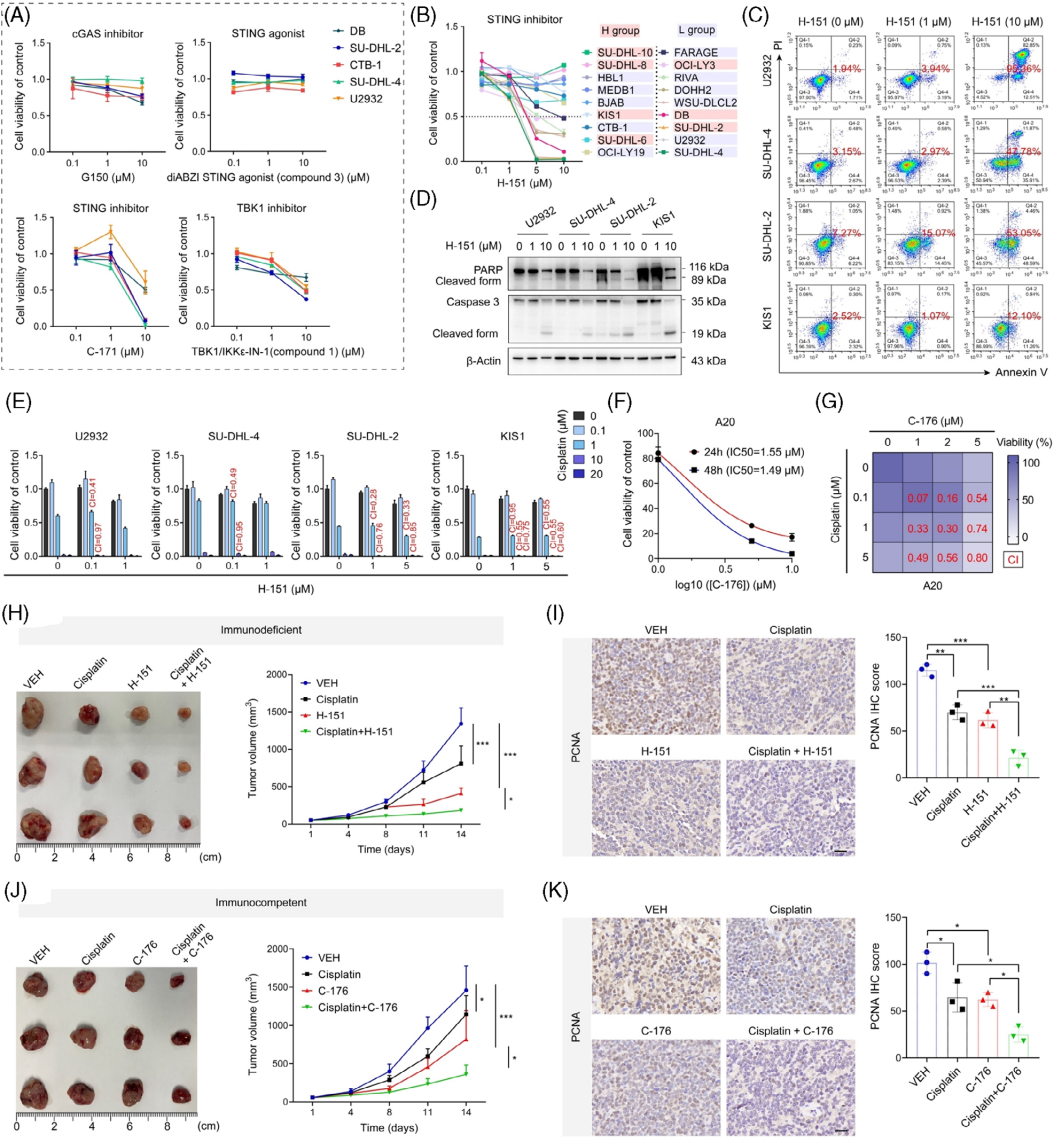
Chemotherapeutic drugs exert synergistic anti‐tumour effects with the inhibitors of STING. (A) The effects of inhibitors and agonists targeting the cGAS‐STING pathway on five representative DLBCL cell lines. (B) CCK8 assays detect the proliferation ability of 18 DLBCL cell lines treated with a STING inhibitor, H‐151, at different concentrations for 48 h. The dashed line represents 0.5. (C) Apoptotic analysis of four representative DLBCL cell lines treated with different concentrations of H‐151 for 48 h. (D) The expression levels of the apoptotic proteins caspase 3, PARP, and their cleaved forms were detected by WB after treatment with different concentrations of H‐151 for 48 h. Representative images of three independent experiments. (E) CCK8 analysis of proliferation activity in U2932, SU‐DHL‐4, SU‐DHL‐2, and KIS1 cell lines treated with H‐151 in combination with DNA‐damaging agents with different concentrations. (F) The inhibitory effect of STING inhibitor C‐176 in A20 cells. (G) The synergistic effect of C‐176 and cisplatin with different concentrations for 24 h. (H) Tumour volume in immunodeficient mice treated with vehicle only (blue), cisplatin (black), H‐151 (red), and H‐151 in combination with cisplatin (green). Three mice in each group. (I) Tumour tissue sections are subjected to IHC for PCNA expression. Scale bar, 20 µm. (J) Tumour volume in immunocompetent mice treated with vehicle only (blue), cisplatin (black), C‐176 (red), and C‐176 in combination with cisplatin (green). Three mice in each group. (K) Tumour tissue sections are subjected to IHC for PCNA expression. Scale bar = 20 µm. All results are expressed as the mean ± SD of three independent experiments. *p*‐values were calculated using two‐way ANOVA (H–K). *p < 0.05, **p < 0.01, ***p < 0.001.

Inhibitors and agonist targeting the cGAS‐STING pathway were then applied to evaluate their roles in DLBCL cells. The inhibition of STING and TBK1 suppressed cell growth dose‐dependently, whereas no significant inhibitory effects were observed with cGAS inhibitors and STING agonists (Figure 5A). Another STING inhibitor, H‐151, was used in more cell lines, and significant inhibitory effects were observed in multiple cell lines (Figure 5B). Subsequently, flow cytometric analysis revealed that H‐151‐induced cell apoptosis and western blotting showed increased levels of activated apoptotic proteins upon treatment (Figure 5C,D). In addition, STING inhibitors H‐151 and C‐176 significantly delayed tumour growth in the immunodeficient and immunocompetent mouse models, respectively (Figure S8A,B). Next, we combined the treatment of H‐151 with DNA‐damaging agents. Notably, the suppression of cell viability was significantly enhanced by combination treatment in most cases (Figure 5E, Figure S8C). C‐176 (a specific inhibitor of STING) also inhibited the proliferation of A20 cells (Figure 5F). A synthetic effect was observed when combined with cisplatin (Figure 5G). Treatment with a TBK1 inhibitor also enhanced the efficacy of cisplatin (Figure S8D). In the in vivo assay, the combination of cisplatin and STING inhibitor H‐151 exerted significantly enhanced anti‐tumour effects compared with the vehicle or single‐agent treatments (Figure 5H). A significant reduction in PCNA levels was observed by the IHC assay, confirming this result (Figure 5I). Immunocompetent mice administered with C‐176 and cisplatin exhibited the slowest tumour growth and lowest PCNA expression levels (Figure 5J,K).

### 3.6 The cGAS/STING expression exhibits heterogeneity and eccDNA abundance is associated with drug sensitivity

**FIGURE 6:**
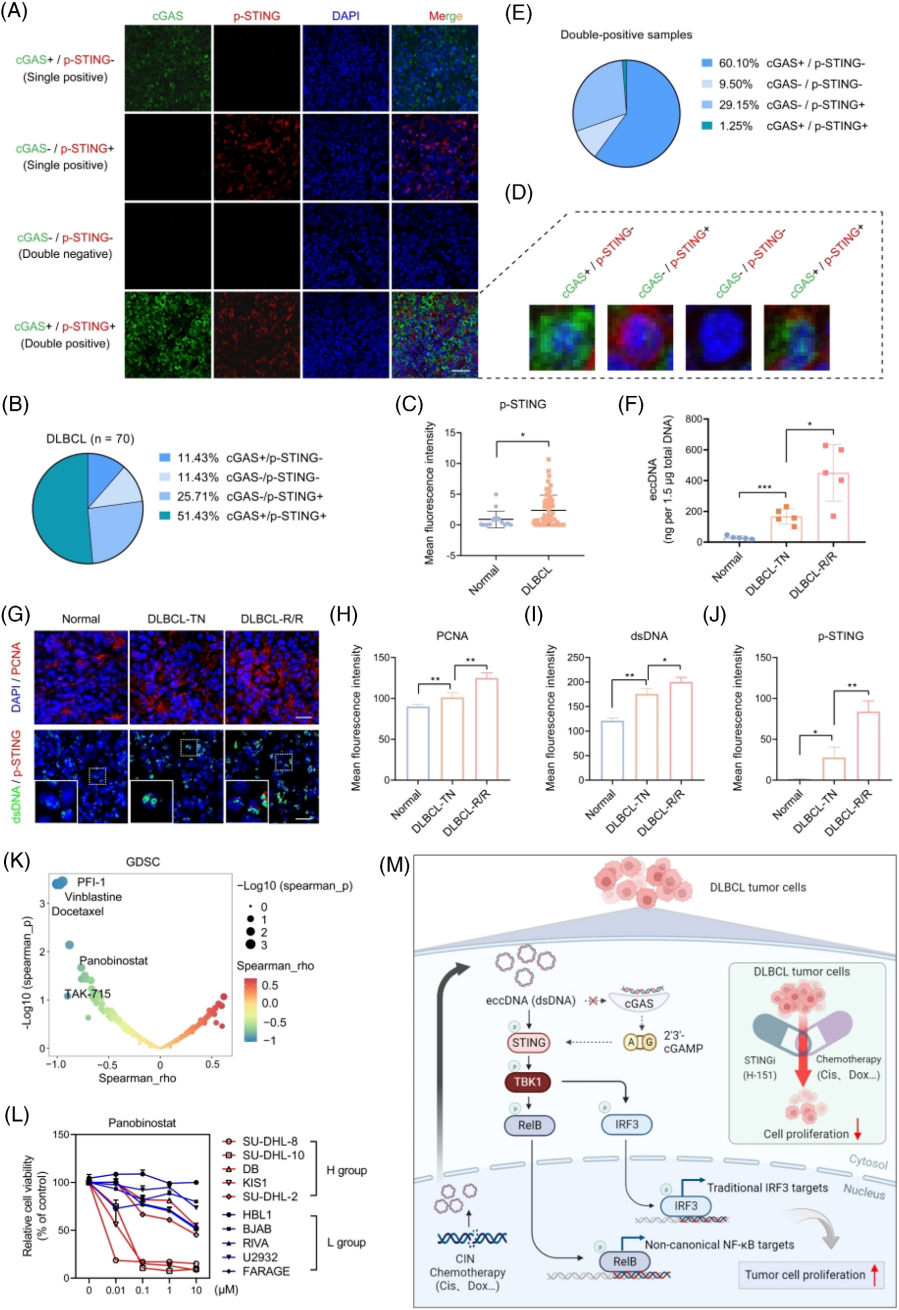
The cGAS/STING expression exhibits heterogeneity and eccDNA abundance is associated with drug sensitivity. (A) The representative IF images of DLBCL patients with or without cGAS and p‐STING expression. Scale bar, 50 µm. (B) The proportion of patients with or without cGAS and p‐STING expression. (C) IF staining shows the levels of p‐STING between normal and DLBCL patients. (D) The representative IF images of cells with or without cGAS and p‐STING expression from double‐positive patients. (E) The proportion of cells with or without cGAS and p‐STING expression in double‐positive patients. (F) Quantification and comparison of eccDNA abundance in tissues from normal patients, treatment‐naïve (TN), and relapsed/refractory (R/R) DLBCL patients. (G) IF assay detects the levels of PCNA, dsDNA, and p‐STING in tissues from normal patients, TN, and R/R DLBCL patients. Scale bar = 20 µm. (H–J) Quantification and comparison of the levels of PCNA (H), dsDNA (I), and p‐STING (J) in three groups. (K) The volcano plot shows the correlation between the IC50 values of drugs from GDSC database and eccDNA abundance in DLBCL cells. (L) CCK8 detects the proliferation ability of five cell lines from H group and five cell lines from L group with the treatment of panobinostat. (M) Schematic representation of eccDNAs induced by DNA damage promoting cell proliferation by activating the STING pathway in a cGAS‐independent manner. All results are expressed as the mean ± SD of three independent experiments. *p*‐values were calculated by two‐tailed Student's *t*‐test (C, F, H–J). *p < 0.05, **p < 0.01, ***p < 0.001.

Differences and heterogeneity in the expression of cGAS and p‐STING were found by the detection of a tissue chip using IF assay composed of 70 DLBCL tumour tissues and 15 normal lymph nodes (Figure S9A). Among the patients, half expressed both cGAS and p‐STING (double‐positive patients), one‐third expressed only one (single‐positive patients), and 11.43 % did not express either (double‐negative patients) (Figure 6A,B). Additionally, p‐STING was significantly upregulated in DLBCL patients, whereas cGAS showed no difference (Figure 6C, Figure S9B). Intriguingly, heterogeneity in the expression of cGAS and p‐STING was also observed in double‐positive patients (Figure 6D). The majority of cells expressed either cGAS or p‐STING, while only 1.25 % of cells expressed both simultaneously (Figure 6E). This finding further supports the notion that cGAS and STING have independent functions in DLBCL, not relying completely on each other. To explore the expression and clinical significance of eccDNAs, we measured their abundance in patient tissues. The qualified data showed that eccDNA abundance was higher in patients with DLBCL compared with normal individuals, particularly in the relapsed and or refractory (R/R) cases (Figure 6F). Furthermore, from the IF assay, we also found the levels of PCNA, dsDNA, and p‐STING were upregulated in DLBCL patients (Figure 6G–J). Notably, p‐STING was found at higher levels in cells with higher dsDNA levels (Figure 6G). In addition to its prognostic implications, eccDNAs are known for contributing to drug resistance.^22^, ^23^ However, it remains unclear whether eccDNAs are involved in drug sensitivity. To address this, we analyzed data from the Genomics of Drug Sensitivity in Cancer (GDSC) database. Of the 345 inhibitors, we identified 9 with IC50 values that showed a negative correlation with the eccDNA abundance (Figure 6K). Subsequently, we selected five inhibitors and confirmed that panobinostat (a potent HDAC inhibitor) showed lower IC50 values in the H group (Figure 6L, Figure S1). These findings imply that the abundance of eccDNAs might affect the sensitivity of tumour cells to drugs.

## 4 DISCUSSION

EccDNAs, as naked DNA, are involved in the progression of various types of cancer.^8^, ^22^, ^24^ It remains to be determined whether eccDNAs actively contribute to the transformation process or whether their presence is a result of genomic instability occurring at a later stage in tumours. According to a recent study, elevated levels of eccDNAs exhibited in precancerous lesions were significantly correlated with the advancement of cancer, suggesting that eccDNAs are an early driver of malignant progression.^25^ Nonetheless, no data to date is available on the expression and roles of eccDNAs in DLBCL. Through the integration of multi‐omics profiling of 18 DLBCL cell lines, we illustrated the existence and characteristics of eccDNAs in DLBCL for the first time and verified their elevated expression in patients. Additionally, we confirmed that cells treated with chemotherapeutic drugs that can result in DNA damage were accompanied by abundant eccDNAs.

As the major regulator in the promotion of metastasis of CIN tumours, the roles of cGAS‐STING are complex and paradoxical.^26^, ^27^, ^28^ Recently, Bakhoum, et al.^29^ explained that the precise mechanism of chronic STING activation induced by CIN contributes to cancer progression. This indicates that eccDNAs may trigger the DNA sensor, activate the cGAS‐STING pathway, and provoke the progression of DLBCL. Indeed, we observed the activation of STING signalling was in correlation with elevated eccDNA abundance in DLBCL. Upregulated p‐STING activated the downstream protein TBK1 and promoted the progression of DLBCL through a non‐classical NF‐κB pathway. Numerous studies have reported the oncogenic roles of TBK1 and NF‐κB components in DLBCL.^30^ TBK1 exhibits high expression levels in DLBCL and is associated with patient prognosis, which also activates the NF‐κB signalling.^31^ Constitutive NF‐κB activation has been shown to be implicated in the tumorigenesis of DLBCL.^32^, ^33^, ^34^ Our study indicates that eccDNAs promote DLBCL progression via the STING/NF‐κB signalling pathway. We investigated the oncogenic functions of STING in DLBCL and its mechanism. Additionally, we discovered that eccDNAs operate in a cGAS‐independent manner, providing further evidence of the independent effects of cGAS and STING beyond the traditional cGAS‐cGAMP‐STING pathway.^35^, ^36^, ^37^

Activating the cGAS‐STING pathway is considered a promising approach for fighting against cancer. For instance, STING agonists have been shown to enhance the effect of monoclonal antibodies for cancer immunotherapy.^38^, ^39^ Furthermore, MIW815 (a STING agonist) has been evaluated in preclinical and phase I clinical trials for advanced/metastatic solid tumours and lymphoma.^40^ Nevertheless, the limited clinical activity of STING agonists was observed, and even some patients experienced tumour progression.^41^ In contrast, C‐176, a STING inhibitor, has been shown to prevent Epstein‐Barr virus (EBV)‐induced B cell transformation and tumorigenesis in EBV‐associated lymphoproliferative disease.^42^ This raises the possibility that blocking STING activation, rather than reinforcing it, may be beneficial for DLBCL patients. In this study, we found that cell proliferation was suppressed by STING and TBK1 inhibitors, not agonists. The anti‐tumour effect of STING inhibitors was more pronounced when combined with chemotherapeutic drugs which can cause DNA damage. The synergy between H‐151 and cisplatin presents an interesting avenue for combinatorial strategies in DLBCL patients, especially those experiencing disease recurrence. DNA damage is revealed to be conducive to the generation of eccDNAs.^21^ For example, Wang's recent research proved the prerequisite role of apoptotic DNA fragmentation in eccDNA generation, as well as the effect of the STING pathway on eccDNA sensing.^43^ In addition, Marco Milán, et al showed that CIN‐induced DNA damage contributed to the leaking of DNA from the nucleus of cancer cells, thereby driving tumour evolution and metastasis.^44^ Similarly, we observed the upregulation of eccDNAs in cells treated with DNA‐damaging agents, also known as chemotherapeutic drugs, which activated the STING signalling. DLBCL is usually accompanied by CIN, which is known to correlate with worse prognosis and therapeutic effectiveness.^45^, ^46^, ^47^ Despite the established safety and efficacy of R‐CHOP, which includes DNA‐damaging drugs, as the current standard for DLBCL treatment, a substantial subset of patients continues to the advent of disease recurrence.^48^ Our findings suggest that eccDNAs induced by intrinsic DNA damage can activate STING signalling in cancer cells, which offers insights into why some DLBCL patients are prone to disease recurrence with the R‐CHOP strategy.

Overall, our study has made the initial discovery that eccDNAs can predict, to some extent, the sensitivity of tumour cells to drugs. Nevertheless, there are specific limitations that need to be acknowledged. For example, further clinical data and pre‐clinical validations are required to explore this potential. At the same time, it is necessary to investigate the specific types of drugs for which sensitivity can be evaluated by the abundance of eccDNAs. Second, considering that PCNA is involved in DNA synthesis and DNA damage tolerance,^49^ and eccDNAs are related to cell proliferation and DNA damage, we inferred that the correlation between PCNA expression and eccDNA abundance may not be coincidental. Whether PCNA or other markers can be used to represent eccDNA abundance, which can be easily detected in a clinical setting, still requires further investigation. Furthermore, the potential use of eccDNA as a prognostic tool in DLBCL requires additional investigation, considering confounding factors like the heterogeneity across molecular subtypes of DLBCL. However, based on the current technological limitations, in this study, we focused on eccDNA abundance and explored its roles and mechanisms in DLBCL. We will devote ourselves to this area, and we believe that, in the future, the concise mechanism underlying the specific type of individual eccDNA or clusters of eccDNAs will be illustrated.

In conclusion, our findings highlight the intriguing role of eccDNAs in tumorigenesis by activating STING signalling in a cGAS‐independent manner (Figure 6M). These results significantly advance our understanding of eccDNAs in DLBCL and provide new insights into the therapeutic strategy for patients.

## AUTHOR CONTRIBUTIONS

Zijuan Wu and Hui Jin designed the experiments. Zijuan Wu, Wei Zhang, and Luqiao Wang performed most of the experiments. Zijuan Wu wrote the manuscript. Hui Jin, Wenyu Shi, Jiayan Leng, and Jianxin Fu supervised the study and revised the manuscript. Jiayan Leng and Jianyong Li assisted with data analysis and performed experiments. Lei Cao and Yongning Jiang collected the samples. Zhou Fan, Mengtao Zhan, Yan Jiang, and Bing Sun analyzed the data. Zijuan Wu, Hui Jin, Wenyu Shi, and Jianyong Li provided funding. All authors contributed to the writing and editing of the manuscript.

## CONFLICT OF INTEREST STATEMENT

The authors declare no conflict of interest.

## ETHICS STATEMENT

All animal experiments were carried out in accordance with the requirements of the Ethics Committee of Nanjing Agricultural University (Permit No. PZW2022033).

## CONSENT FOR PUBLICATION

The authors agree to the publication of all the data involved in this article.

## Supporting information

Supporting information

Supporting information

## Data Availability

The raw sequence data reported in this paper have been deposited in the Genome Sequence Archive (Genomics, Proteomics & Bioinformatics 2021) in National Genomics Data Center (Nucleic Acids Res 2022), China National Center for Bioinformation/Beijing Institute of Genomics, Chinese Academy of Sciences (GSA‐Human: HRA005445) that are publicly accessible at https://ngdc.cncb.ac.cn/gsa‐human. Further information should be directed to and will be answered by the corresponding author.

## References

[1] Sehn LH , Salles G . Diffuse large B‐cell lymphoma. N Engl J Med. 2021;384(9):842‐858. doi:10.1056/NEJMra2027612 33657296 PMC8377611

[2] Wästerlid T , Harrysson S , Andersson TM , et al. Outcome and determinants of failure to complete primary R‐CHOP treatment for reasons other than non‐response among patients with diffuse large B‐cell lymphoma. Am J Hematol. 2020;95(7):740‐748. doi:10.1002/ajh.25789 32180274

[3] Friedberg JW . Relapsed/refractory diffuse large B‐cell lymphoma. Hematology (Am Soc Hematol Educ Program). 2011;2011:498‐505. doi:10.1182/asheducation-2011.1.498 22160081

[4] Shao R , Liu C , Xue R , et al. Tumor‐derived exosomal ENO2 modulates polarization of tumor‐associated macrophages through reprogramming glycolysis to promote progression of diffuse large B‐cell lymphoma. Int J Biol Sci. 2024;20(3):848‐863. doi:10.7150/ijbs.91154 38250157 PMC10797692

[5] Wang H , Shao R , Liu W , Tang H , Lu Y . Identification of a prognostic metabolic gene signature in diffuse large B‐cell lymphoma. J Cell Mol Med. 2021;25(14):7066‐7077. doi:10.1111/jcmm.16720 34128320 PMC8278125

[6] Abbasi AF , Asim MN , Ahmed S , Dengel A . Long extrachromosomal circular DNA identification by fusing sequence‐derived features of physicochemical properties and nucleotide distribution patterns. Sci Rep. 2024;14(1):9466. doi:10.1038/s41598-024-57457-5 38658614 PMC11043385

[7] Vogt N , Gibaud A , Lemoine F , de la Grange P , Debatisse M , Malfoy B . Amplicon rearrangements during the extrachromosomal and intrachromosomal amplification process in a glioma. Nucleic Acids Res. 2014;42(21):13194‐131205. doi:10.1093/nar/gku1101 25378339 PMC4245956

[8] Turner KM , Deshpande V , Beyter D , et al. Extrachromosomal oncogene amplification drives tumour evolution and genetic heterogeneity. Nature. 2017;543(7643):122‐125. doi:10.1038/nature21356 28178237 PMC5334176

[9] Yi E , Gujar AD , Guthrie M , et al. Live‐cell imaging shows uneven segregation of extrachromosomal DNA elements and transcriptionally active extrachromosomal DNA hubs in cancer. Cancer Discov. 2022;12(2):468‐483. doi:10.1158/2159-8290.Cd-21-1376 34819316 PMC8831456

[10] Li R , Wang Y , Li J , Zhou X . Extrachromosomal circular DNA (eccDNA): an emerging star in cancer. Biomark Res. 2022;10(1):53. doi:10.1186/s40364-022-00399-9 35883211 PMC9327165

[11] Deshpande V , Luebeck J , Nguyen ND , et al. Exploring the landscape of focal amplifications in cancer using AmpliconArchitect. Nat Commun. 2019;10(1):392. doi:10.1038/s41467-018-08200-y 30674876 PMC6344493

[12] Ling X , Han Y , Meng J , et al. Small extrachromosomal circular DNA (eccDNA): major functions in evolution and cancer. Mol Cancer. 2021;20(1):113. doi:10.1186/s12943-021-01413-8 34479546 PMC8414719

[13] Hopfner KP , Hornung V . Molecular mechanisms and cellular functions of cGAS‐STING signalling. Nat Rev Mol Cell Biol. 2020;21(9):501‐521. doi:10.1038/s41580-020-0244-x 32424334

[14] Decout A , Katz JD , Venkatraman S , Ablasser A . The cGAS‐STING pathway as a therapeutic target in inflammatory diseases. Nat Rev Immunol. 2021;21(9):548‐569. doi:10.1038/s41577-021-00524-z 33833439 PMC8029610

[15] Samson N , Ablasser A . The cGAS‐STING pathway and cancer. Nat Cancer. 2022;3(12):1452‐1463. doi:10.1038/s43018-022-00468-w 36510011

[16] Kwon J , Bakhoum SF . The Cytosolic DNA‐Sensing cGAS‐STING Pathway in Cancer. Cancer Discov. 2020;10(1):26‐39. doi:10.1158/2159-8290.Cd-19-0761 31852718 PMC7151642

[17] Ka NL , Park MK , Kim SS , et al. NR1D1 stimulates antitumor immune responses in breast cancer by activating cGAS‐STING signaling. Cancer Res. 2023. doi:10.1158/0008-5472.Can-23-0329 PMC10538367 37395684

[18] Liu H , Zhang H , Wu X , et al. Nuclear cGAS suppresses DNA repair and promotes tumorigenesis. Nature. 2018;563(7729):131‐136. doi:10.1038/s41586-018-0629-6 30356214

[19] Bakhoum SF , Ngo B , Laughney AM , et al. Chromosomal instability drives metastasis through a cytosolic DNA response. Nature. 2018;553(7689):467‐472. doi:10.1038/nature25432 29342134 PMC5785464

[20] Hu J , Sánchez‐Rivera FJ , Wang Z , et al. STING inhibits the reactivation of dormant metastasis in lung adenocarcinoma. Nature. 2023;616(7958):806‐813. doi:10.1038/s41586-023-05880-5 36991128 PMC10569211

[21] Dillon LW , Kumar P , Shibata Y , et al. Production of extrachromosomal MicroDNAs is linked to mismatch repair pathways and transcriptional activity. Cell Rep. 2015;11(11):1749‐1759. doi:10.1016/j.celrep.2015.05.020 26051933 PMC4481157

[22] Kim H , Nguyen NP , Turner K , et al. Extrachromosomal DNA is associated with oncogene amplification and poor outcome across multiple cancers. Nat Genet. 2020;52(9):891‐897. doi:10.1038/s41588-020-0678-2 32807987 PMC7484012

[23] Nathanson DA , Gini B , Mottahedeh J , et al. Targeted therapy resistance mediated by dynamic regulation of extrachromosomal mutant EGFR DNA. Science. 2014;343(6166):72‐76. doi:10.1126/science.1241328 24310612 PMC4049335

[24] Nikolaev S , Santoni F , Garieri M , et al. Extrachromosomal driver mutations in glioblastoma and low‐grade glioma. Nat Commun. 2014;5:5690. doi:10.1038/ncomms6690 25471132 PMC4338529

[25] Luebeck J , Ng AWT , Galipeau PC , et al. Extrachromosomal DNA in the cancerous transformation of Barrett's oesophagus. Nature. 2023;616(7958):798‐805. doi:10.1038/s41586-023-05937-5 37046089 PMC10132967

[26] Chen T , Xu ZG , Luo J , et al. NSUN2 is a glucose sensor suppressing cGAS/STING to maintain tumorigenesis and immunotherapy resistance. Cell Metab. 2023;35(10):1782‐1798. doi:10.1016/j.cmet.2023.07.009 e8 37586363 PMC10726430

[27] Frittoli E , Palamidessi A , Iannelli F , et al. Tissue fluidification promotes a cGAS‐STING cytosolic DNA response in invasive breast cancer. Nat Mater. 2023;22(5):644‐655. doi:10.1038/s41563-022-01431-x 36581770 PMC10156599

[28] Gulen MF , Samson N , Keller A , et al. cGAS‐STING drives ageing‐related inflammation and neurodegeneration. Nature. 2023;620(7973):374‐380. doi:10.1038/s41586-023-06373-1 37532932 PMC10412454

[29] Li J , Hubisz MJ , Earlie EM , et al. Non‐cell‐autonomous cancer progression from chromosomal instability. Nature. 2023. doi:10.1038/s41586-023-06464-z PMC10468402 37612508

[30] Eluard B , Nuan‐Aliman S , Faumont N , et al. The alternative RelB NF‐κB subunit is a novel critical player in diffuse large B‐cell lymphoma. Blood. 2022;139(3):384‐398. doi:10.1182/blood.2020010039 34232979

[31] Carr M , Mamand S , Chapman KL , Perrior T , Wagner SD . IKKε and TBK1 in diffuse large B‐cell lymphoma: a possible mechanism of action of an IKKε/TBK1 inhibitor to repress NF‐κB and IL‐10 signalling. J Cell Mol Med. 2020;24(19):11573‐11582. doi:10.1111/jcmm.15774 32858764 PMC7576278

[32] Zhao Q , Fu W , Jiang H , et al. Clinicopathological implications of nuclear factor κB signal pathway activation in diffuse large B‐cell lymphoma. Hum Pathol. 2015;46(4):524‐531. doi:10.1016/j.humpath.2014.06.032 25636172

[33] Ngo VN , Young RM , Schmitz R , et al. Oncogenically active MYD88 mutations in human lymphoma. Nature. 2011;470(7332):115‐119. doi:10.1038/nature09671 21179087 PMC5024568

[34] Esmeray E , Küçük C . Genetic alterations in B cell lymphoma subtypes as potential biomarkers for noninvasive diagnosis, prognosis, therapy, and disease monitoring. Turk J Biol = Turk biyoloji dergisi. 2020;44(1):1‐14. doi:10.3906/biy-1908-23 32123491 PMC7049453

[35] Li K , Liu Y , Xu Z , et al. Avian oncogenic herpesvirus antagonizes the cGAS‐STING DNA‐sensing pathway to mediate immune evasion. PLoS Pathog. 2019;15(9):e1007999. doi:10.1371/journal.ppat.1007999 31539404 PMC6799934

[36] Long Y , Guo J , Chen J , et al. GPR162 activates STING dependent DNA damage pathway as a novel tumor suppressor and radiation sensitizer. Signal Transduct Targeted Ther. 2023;8(1):48. doi:10.1038/s41392-022-01224-3 PMC9892510 36725837

[37] Dunphy G , Flannery SM , Almine JF , et al. Non‐canonical activation of the DNA sensing adaptor STING by ATM and IFI16 mediates NF‐κB signaling after nuclear DNA damage. Mol Cell. 2018;71(5):745‐760. doi:10.1016/j.molcel.2018.07.034 e5 30193098 PMC6127031

[38] Dahal LN , Dou L , Hussain K , et al. STING activation reverses lymphoma‐mediated resistance to antibody immunotherapy. Cancer Res. 2017;77(13):3619‐3631. doi:10.1158/0008-5472.Can-16-2784 28512240 PMC5500176

[39] Luo J , Pang S , Hui Z , et al. Blocking Tim‐3 enhances the anti‐tumor immunity of STING agonist ADU‐S100 by unleashing CD4^+^T cells through regulating type 2 conventional dendritic cells. Theranostics. 2023;13(14):4836‐4857. doi:10.7150/thno.86792 37771774 PMC10526657

[40] Meric‐Bernstam F , Sweis RF , Hodi FS , et al. Phase I dose‐escalation trial of MIW815 (ADU‐S100), an intratumoral STING agonist, in patients with advanced/metastatic solid tumors or lymphomas. Clin Cancer Res. 2022;28(4):677‐688. doi:10.1158/1078-0432.Ccr-21-1963 34716197

[41] Meric‐Bernstam F , Sweis RF , Kasper S , et al. Combination of the STING agonist MIW815 (ADU‐S100) and PD‐1 inhibitor spartalizumab in advanced/metastatic solid tumors or lymphomas: an open‐label, multicenter, phase Ib study. Clin Cancer Res. 2023;29(1):110‐121. doi:10.1158/1078-0432.Ccr-22-2235 36282874 PMC11188043

[42] Miyagi S , Watanabe T , Hara Y , et al. A STING inhibitor suppresses EBV‐induced B cell transformation and lymphomagenesis. Cancer Sci. 2021;112(12):5088‐5099. doi:10.1111/cas.15152 34609775 PMC8645724

[43] Wang Y , Wang M , Djekidel MN , et al. eccDNAs are apoptotic products with high innate immunostimulatory activity. Nature. 2021;599(7884):308‐314. doi:10.1038/s41586-021-04009-w 34671165 PMC9295135

[44] Barrio L , Gaspar AE , Muzzopappa M , et al. Chromosomal instability‐induced cell invasion through caspase‐driven DNA damage. Curr Biol. 2023. doi:10.1016/j.cub.2023.09.004 37751744

[45] Bakhoum SF , Danilova OV , Kaur P , Levy NB , Compton DA . Chromosomal instability substantiates poor prognosis in patients with diffuse large B‐cell lymphoma. Clin Cancer Res. 2011;17(24):7704‐7711. doi:10.1158/1078-0432.Ccr-11-2049 22184286 PMC3244806

[46] Kim DY , Nam J , Chung JS , et al. Predictive parameters of febrile neutropenia and clinical significance of G‐CSF receptor signaling pathway in the development of neutropenia during R‐CHOP chemotherapy with prophylactic pegfilgrastim in patients with diffuse large B‐Cell lymphoma. Cancer Res Treat. 2022;54(4):1256‐1267. doi:10.4143/crt.2021.944 34990523 PMC9582470

[47] Nanjangud G , Rao PH , Hegde A , et al. Spectral karyotyping identifies new rearrangements, translocations, and clinical associations in diffuse large B‐cell lymphoma. Blood. 2002;99(7):2554‐2561. doi:10.1182/blood.v99.7.2554 11895793

[48] Poletto S , Novo M , Paruzzo L , Frascione PMM , Vitolo U . Treatment strategies for patients with diffuse large B‐cell lymphoma. Cancer Treat Rev. 2022;110:102443. doi:10.1016/j.ctrv.2022.102443 35933930

[49] Zhang S , Zhou T , Wang Z , et al. Post‐translational modifications of PCNA in control of DNA synthesis and DNA damage tolerance‐the implications in carcinogenesis. Int J Biol Sci. 2021;17(14):4047‐4059. doi:10.7150/ijbs.64628 34671219 PMC8495385

