# Erratum for the Clinical and Translational Medicine “Multi‐omics integration reveals the oncogenic role of eccDNAs in diffuse large B‐cell lymphoma through STING signalling” by Zijuan Wu et al.

**DOI:** 10.1002/ctm2.70301

**Published:** 2025-04-01

**Authors:** Zijuan Wu, Wei Zhang, Luqiao Wang, Jiayan Leng, Yongle Li, Zhou Fan, Mengtao Zhan, Lei Cao, Yongning Jiang, Yan Jiang, Bing Sun, Jianxin Fu, Jianyong Li, Wenyu Shi, Hui Jin

**Affiliations:** ^1^ Lymphoma Center Department of Hematology the First Affiliated Hospital of Nanjing Medical University Jiangsu Province Hospital Nanjing Medical University Nanjing China; ^2^ Key Laboratory of Hematology of Nanjing Medical University Nanjing China; ^3^ Jiangsu Key Lab of Cancer Biomarkers Prevention and Treatment Collaborative Innovation Center for Personalized Cancer Medicine Nanjing Medical University Nanjing China; ^4^ Department of Hematology Suqian Hospital Jiangsu Province Hospital Suqian China; ^5^ Department of Hematology Sir Run Run Shaw Hospital Zhejiang University School of Medicine Hangzhou China; ^6^ Department of Hematology Affiliated People's Hospital of Jiangsu University Zhenjiang China; ^7^ Department of Oncology Affiliated Hospital of Nantong University Nantong China; ^8^ The Central Research Laboratory The First Affiliated Hospital of Soochow University Suzhou China

Zijuan Wu. Clin Transl Med. 2024;14(8):e1807.

Following the publication of the original article,^1^ the authors identified minor errors in Figure 1C, where the images of one group was incorrect. Because during the image acquisition, we mistakenly labelled two duplicate results from a single sample. We have made the necessary corrections to Figure 1C. More importantly, we promise that the erratum has no impact on the conclusion and description of the article.

**FIGURE 1:**
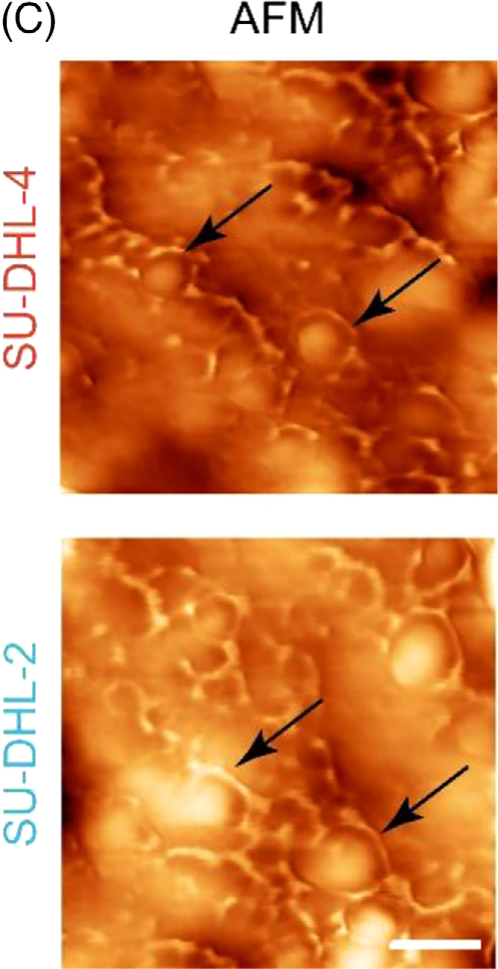
(C) AFM images of extracted eccDNAs in DLBCL cell lines. Scale bar, 200 nm. UPDATED FIGURE 1. (C) AFM images of extracted eccDNAs in DLBCL cell lines. Scale bar, 200 nm.

We apologize for this error.
