# Erratum for the Clinical and Translational Medicine “Multi‐omics integration reveals the oncogenic role of eccDNAs in diffuse large B‐cell lymphoma through STING signalling” by Zijuan Wu et al.

**DOI:** 10.1002/ctm2.70312

**Published:** 2025-07-24

Following the publication of the original article,^1^ the authors identified minor errors in Figure 1C, where the images of one group were incorrect. Because during the image acquisition, we mistakenly labelled two duplicate results from a single sample. We have made the necessary corrections to Figure 1C. More importantly, we promise that the erratum has no impact on the conclusion and description of the article.

We apologise for this error.

ORIGINAL FIGURE 1.


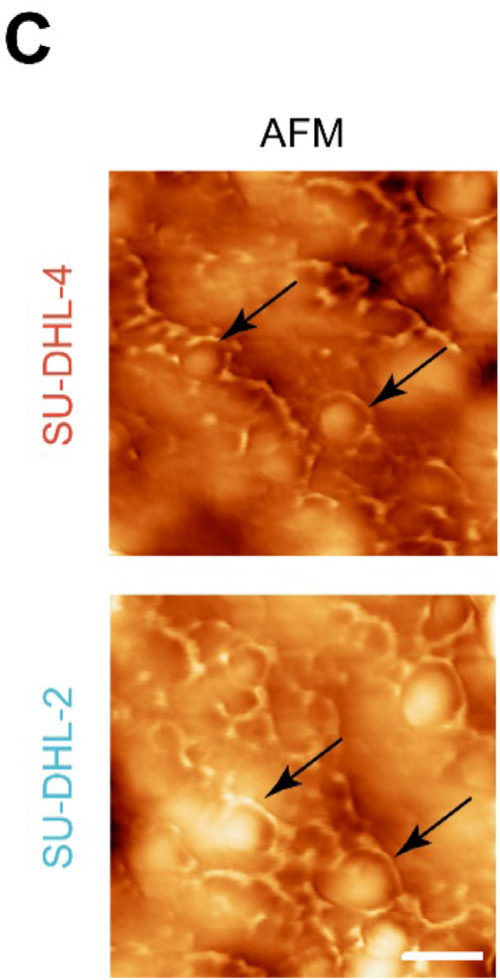


(C) AFM images of extracted eccDNAs in DLBCL cell lines. Scale bar, 200 nm.

UPDATED FIGURE 1.


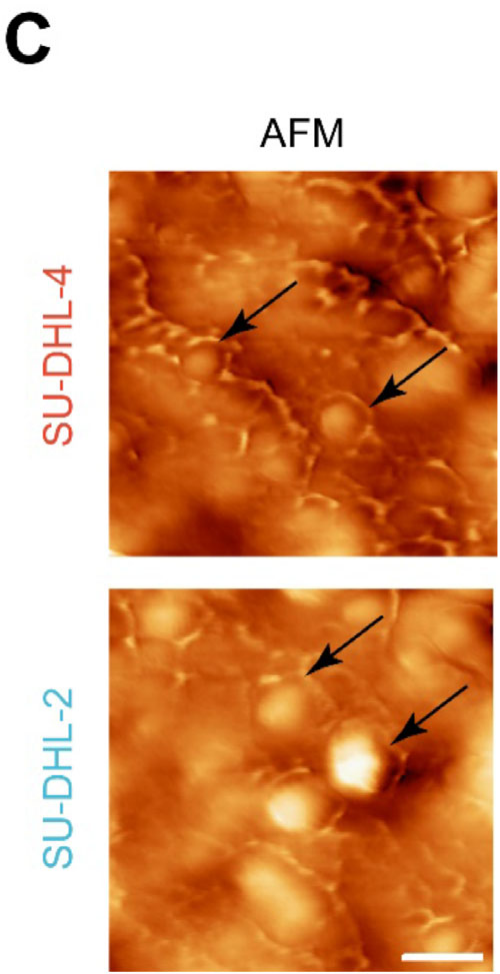


(C) AFM images of extracted eccDNAs in DLBCL cell lines. Scale bar, 200 nm.

1. Wu Z, Zhang W, Wang L, et al. Multi‐omics integration reveals the oncogenic role of eccDNAs in diffuse large B‐cell lymphoma through STING signalling. *Clin Transl Med*. 2024;14(8):e1815 https://doi.org/10.1002/ctm2.1815.

