# Supplementary material for: Multi‐omics integration reveals the oncogenic role of eccDNAs in diffuse large B‐cell lymphoma through STING signalling
Source: Clin Transl Med. 2024 Aug 25;14(8):e1815. doi: 10.1002/ctm2.1815 (PMC11345442; doi:10.1002/ctm2.1815)
Supplement: Supplementary file 1 — Supporting information [file CTM2-14-e1815-s001.pdf]

Figure S1

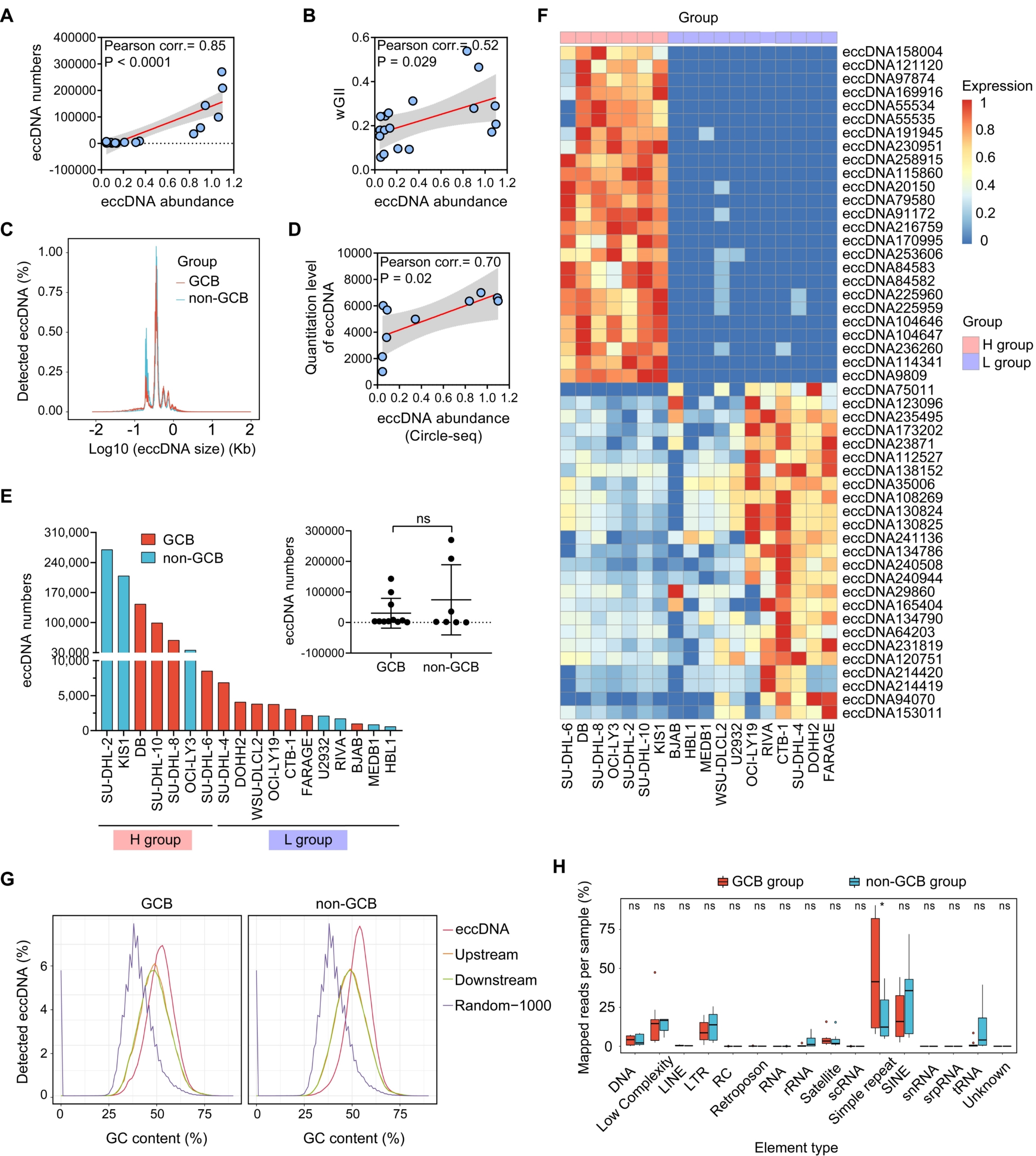

A
Pearson corr.= 0.85
P < 0.0001
eccDNA numbers
eccDNA abundance
B
Pearson corr.= 0.52
P = 0.029
wGII
eccDNA abundance
C
Group
GCB
non-GCB
Detected eccDNA (%)
Log10 (eccDNA size) (Kb)
D
Pearson corr.= 0.70
P = 0.02
Quantitation level of eccDNA
eccDNA abundance (Circle-seq)
E
GCB
non-GCB
eccDNA numbers
SU-DHL-2
KIS1
DB
SU-DHL-10
SU-DHL-8
OCI-LY3
SU-DHL-6
SU-DHL-4
DOHH2
WSU-DLCL2
OCI-LY19
CTB-1
FARAGE
U2932
RIVA
BJAB
MEDB1
HBL1
H group
L group
ns
F
Group
Expression
H group
L group
eccDNA158004
eccDNA121120
eccDNA97874
eccDNA169916
eccDNA55534
eccDNA55535
eccDNA191945
eccDNA230951
eccDNA258915
eccDNA115860
eccDNA20150
eccDNA79580
eccDNA91172
eccDNA216759
eccDNA170995
eccDNA253606
eccDNA84583
eccDNA84582
eccDNA225960
eccDNA225959
eccDNA104646
eccDNA104647
eccDNA236260
eccDNA114341
eccDNA9809
eccDNA75011
eccDNA123096
eccDNA235495
eccDNA173202
eccDNA23871
eccDNA112527
eccDNA138152
eccDNA35006
eccDNA108269
eccDNA130824
eccDNA130825
eccDNA241136
eccDNA134786
eccDNA240508
eccDNA240944
eccDNA29860
eccDNA165404
eccDNA134790
eccDNA64203
eccDNA231819
eccDNA120751
eccDNA214420
eccDNA214419
eccDNA94070
eccDNA153011
G
GCB
non-GCB
eccDNA
Upstream
Downstream
Random−1000
Detected eccDNA (%)
GC content (%)
H
GCB group
non-GCB group
Mapped reads per sample (%)
DNA
Low Complexity
LINE
LTR
RC
Retroposon
RNA
rRNA
Satellite
scRNA
Simple repeat
SINE
snRNA
srpRNA
tRNA
Unknown
Element type

Figure S2

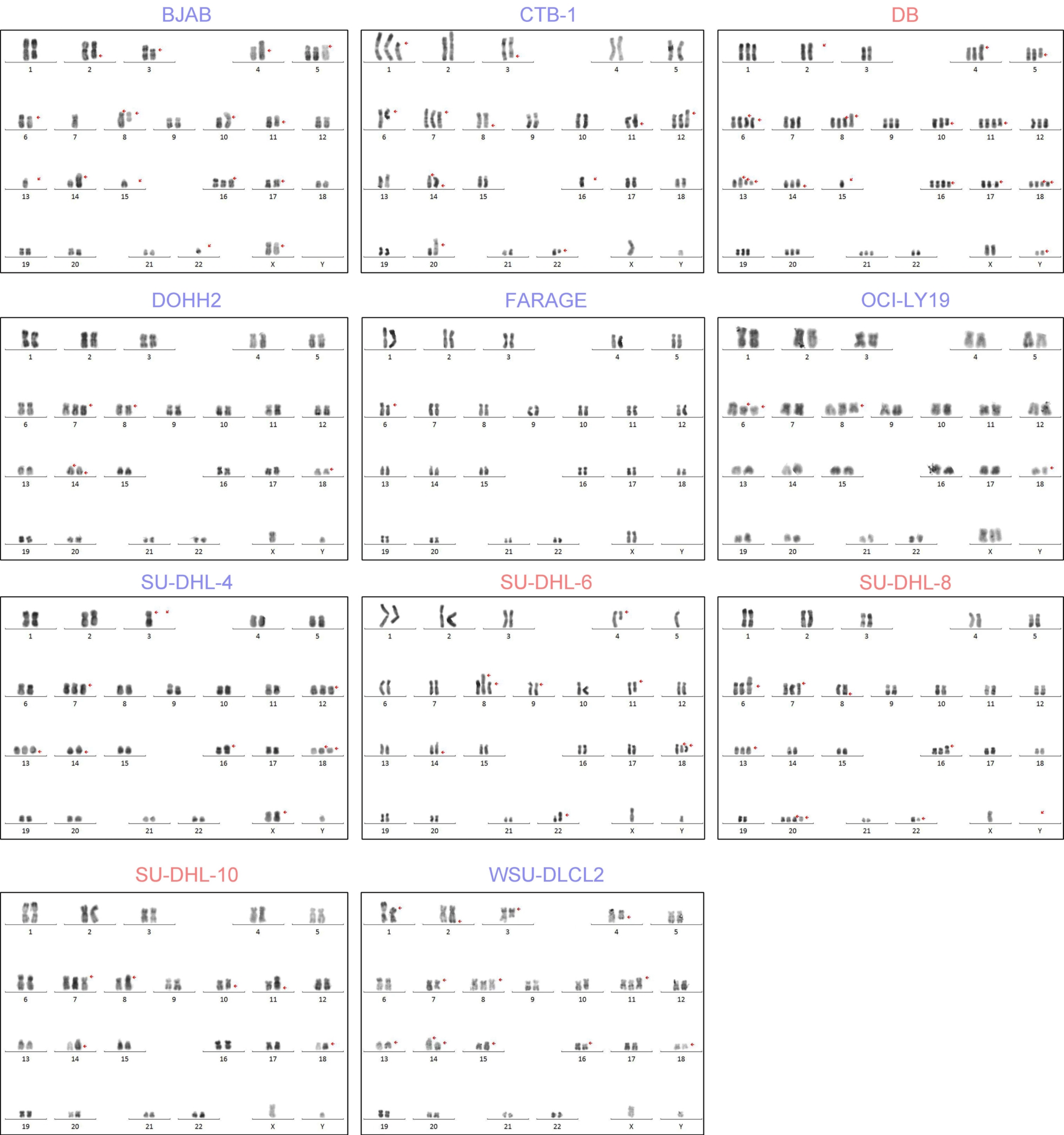
BJAB
CTB-1
DB
DOHH2
FARAGE
OCI-LY19
SU-DHL-4
SU-DHL-6
SU-DHL-8
SU-DHL-10
WSU-DLCL2

Figure S3

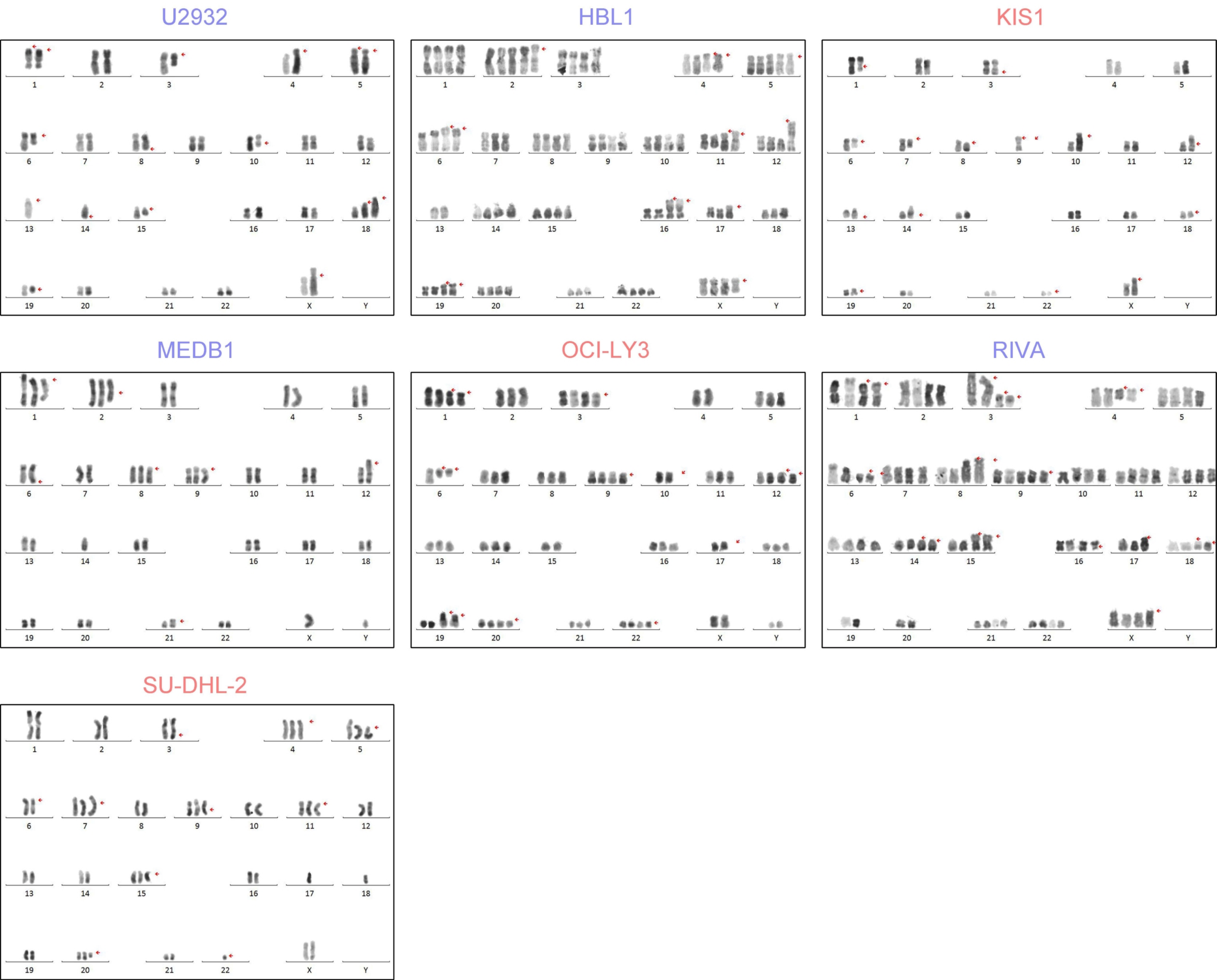

Figure S4

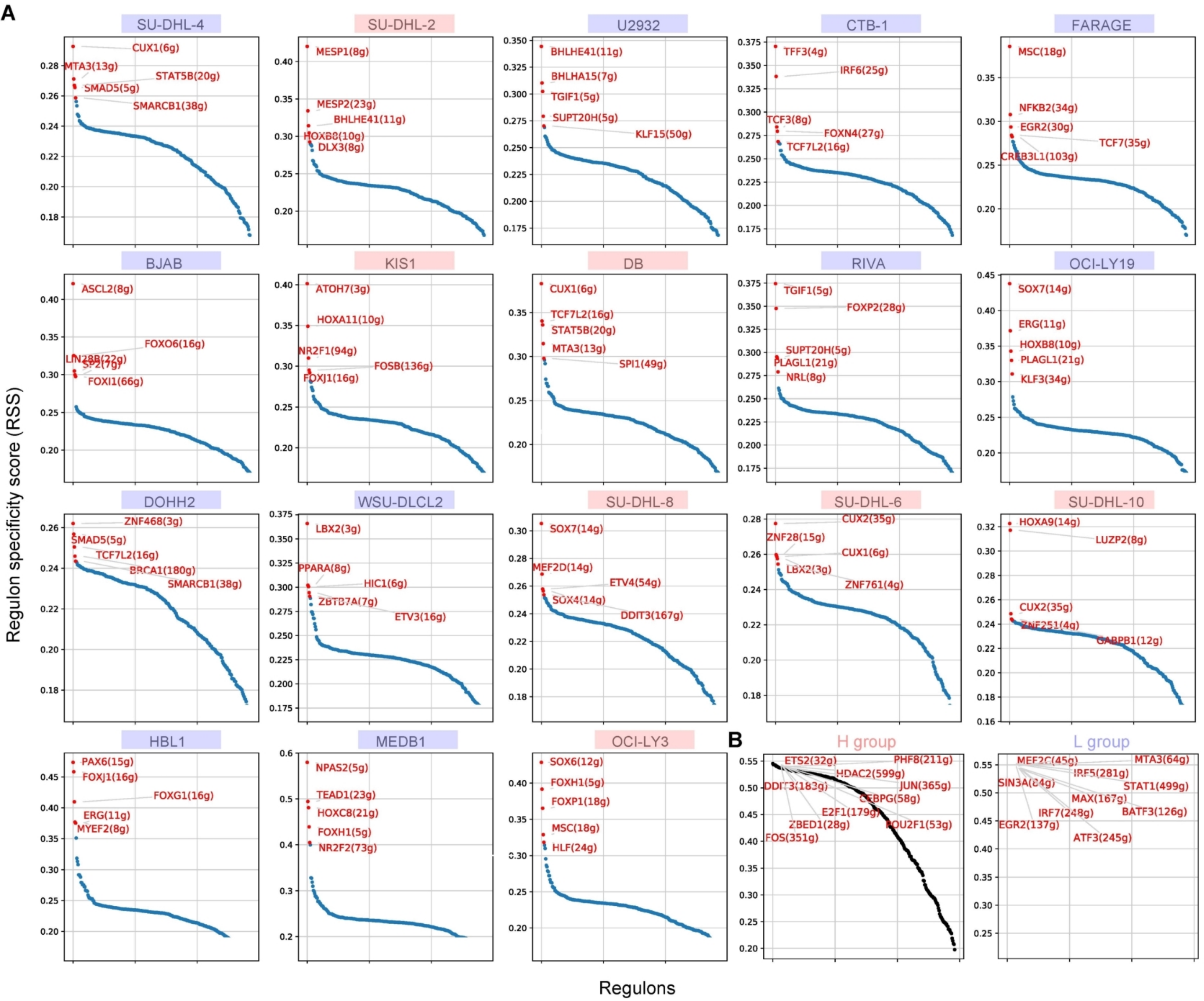

A
SU-DHL-4
CUX1(6g)
MTA3(13g)
STAT5B(20g)
SMAD5(5g)
SMARCB1(38g)
SU-DHL-2
MESP1(8g)
MESP2(23g)
BHLHE41(11g)
HOXB8(10g)
DLX3(8g)
U2932
BHLHE41(11g)
BHLHA15(7g)
TGIF1(5g)
SUPT20H(5g)
KLF15(50g)
CTB-1
TFF3(4g)
IRF6(25g)
TCF3(8g)
FOXN4(27g)
TCF7L2(16g)
FARAGE
MSC(18g)
NFKB2(34g)
EGR2(30g)
TCF7(35g)
CREB3L1(103g)
BJAB
ASCL2(8g)
FOXO6(16g)
LIN28B(22g)
SP2(7g)
FOXI1(66g)
KIS1
ATOH7(3g)
HOXA11(10g)
NR2F1(94g)
FOSB(136g)
FOXJ1(16g)
DB
CUX1(6g)
TCF7L2(16g)
STAT5B(20g)
MTA3(13g)
SPI1(49g)
RIVA
TGIF1(5g)
FOXP2(28g)
SUPT20H(5g)
PLAGL1(21g)
NRL(8g)
OCI-LY19
SOX7(14g)
ERG(11g)
HOXB8(10g)
PLAGL1(21g)
KLF3(34g)
DOHH2
ZNF468(3g)
SMAD5(5g)
TCF7L2(16g)
BRCA1(180g)
SMARCB1(38g)
WSU-DLCL2
LBX2(3g)
PPARA(8g)
HIC1(6g)
ZBTB7A(7g)
ETV3(16g)
SU-DHL-8
SOX7(14g)
MEF2D(14g)
ETV4(54g)
SOX4(14g)
DDIT3(167g)
SU-DHL-6
CUX2(35g)
ZNF28(15g)
CUX1(6g)
LBX2(3g)
ZNF761(4g)
SU-DHL-10
HOXA9(14g)
LUZP2(8g)
CUX2(35g)
ZNF251(4g)
GABPB1(12g)
HBL1
PAX6(15g)
FOXJ1(16g)
FOXG1(16g)
ERG(11g)
MYEF2(8g)
MEDB1
NPAS2(5g)
TEAD1(23g)
HOXC8(21g)
FOXH1(5g)
NR2F2(73g)
OCI-LY3
SOX6(12g)
FOXH1(5g)
FOXP1(18g)
MSC(18g)
HLF(24g)
B
H group
ETS2(32g)
PHF8(211g)
HDAC2(599g)
DDIT3(183g)
JUN(365g)
CEBPG(58g)
E2F1(179g)
ZBED1(28g)
POU2F1(53g)
FOS(351g)
L group
MEF2C(45g)
MTA3(64g)
IRF5(281g)
SIN3A(84g)
STAT1(499g)
MAX(167g)
IRF7(248g)
BATF3(126g)
EGR2(137g)
ATF3(245g)
Regulon specificity score (RSS)
Regulons

Figure S5

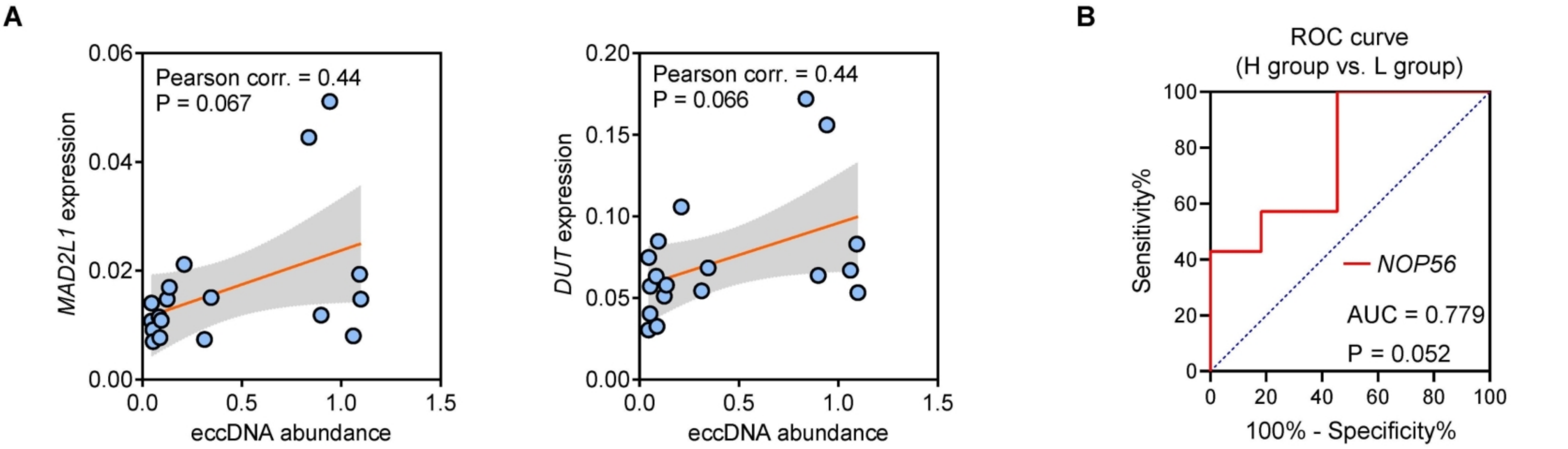

A
Pearson corr. = 0.44
P = 0.067
MAD2L1 expression
0.06
0.04
0.02
0.00
0.0
0.5
1.0
1.5
eccDNA abundance
Pearson corr. = 0.44
P = 0.066
DUT expression
0.20
0.15
0.10
0.05
0.00
0.0
0.5
1.0
1.5
eccDNA abundance
B
ROC curve
(H group vs. L group)
Sensitivity%
100
80
60
40
20
0
NOP56
AUC = 0.779
P = 0.052
0
20
40
60
80
100
100% - Specificity%

Figure S6

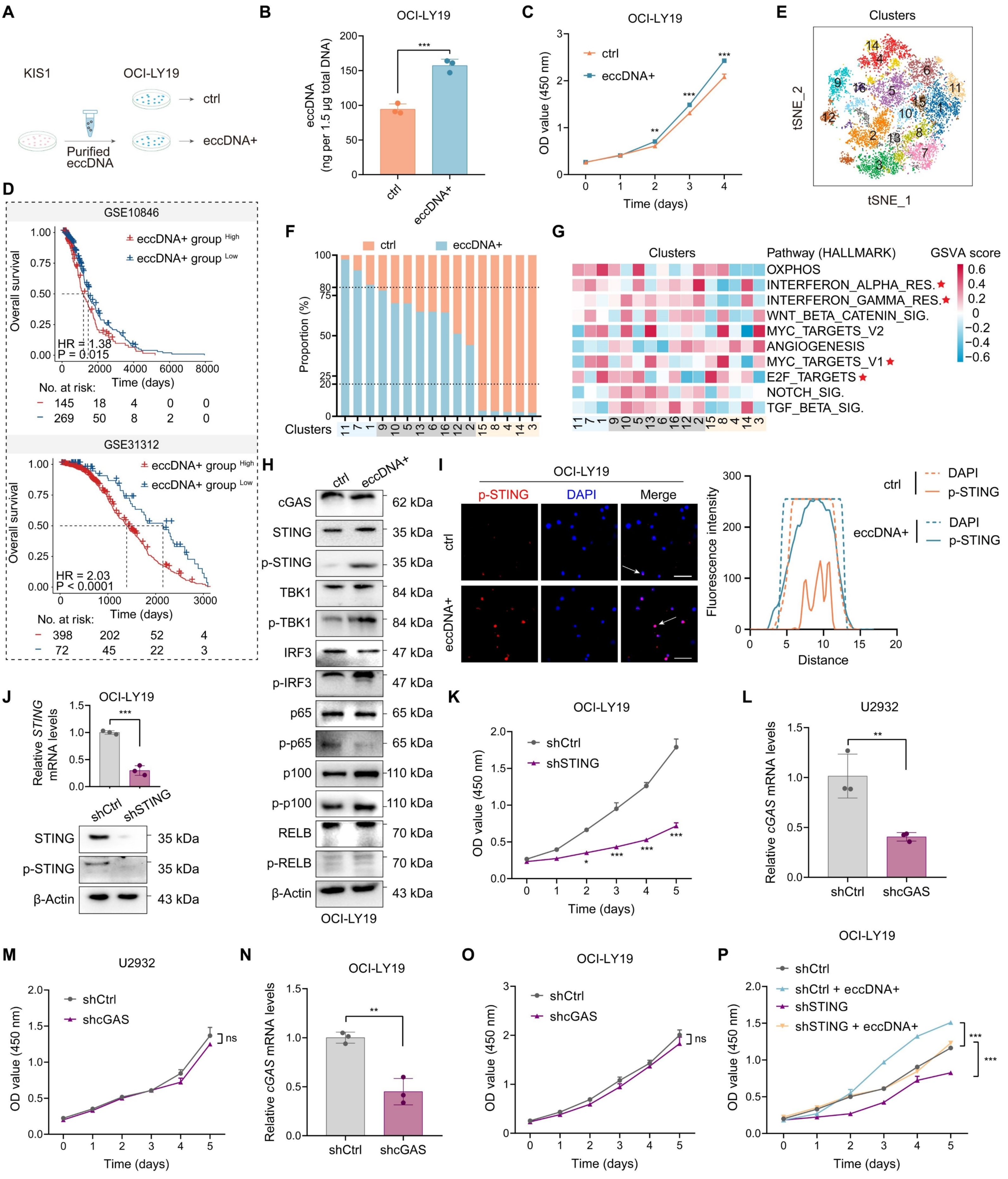

A
KIS1
OCI-LY19
Purified eccDNA
ctrl
eccDNA+
B
OCI-LY19
eccDNA (ng per 1.5 μg total DNA)
ctrl
eccDNA+
***
C
OCI-LY19
OD value (450 nm)
Time (days)
ctrl
eccDNA+
E
Clusters
tSNE_1
tSNE_2
D
GSE10846
Overall survival
eccDNA+ group High
eccDNA+ group Low
HR = 1.38
P = 0.015
Time (days)
No. at risk:
145 18 4 0 0
269 50 8 2 0
GSE31312
HR = 2.03
P < 0.0001
No. at risk:
398 202 52 4
72 45 22 3
F
ctrl
eccDNA+
Proportion (%)
Clusters
G
Clusters
Pathway (HALLMARK)
GSVA score
OXPHOS
INTERFERON_ALPHA_RES.
INTERFERON_GAMMA_RES.
WNT_BETA_CATENIN_SIG.
MYC_TARGETS_V2
ANGIOGENESIS
MYC_TARGETS_V1
E2F_TARGETS
NOTCH_SIG.
TGF_BETA_SIG.
H
ctrl
eccDNA+
cGAS 62 kDa
STING 35 kDa
p-STING 35 kDa
TBK1 84 kDa
p-TBK1 84 kDa
IRF3 47 kDa
p-IRF3 47 kDa
p65 65 kDa
p-p65 65 kDa
p100 110 kDa
p-p100 110 kDa
RELB 70 kDa
p-RELB 70 kDa
β-Actin 43 kDa
OCI-LY19
I
OCI-LY19
p-STING
DAPI
Merge
ctrl
eccDNA+
Fluorescence intensity
Distance
DAPI
p-STING
J
OCI-LY19
Relative STING mRNA levels
shCtrl
shSTING
STING 35 kDa
p-STING 35 kDa
β-Actin 43 kDa
K
OCI-LY19
OD value (450 nm)
Time (days)
shCtrl
shSTING
L
U2932
Relative cGAS mRNA levels
shCtrl
shcGAS
M
U2932
OD value (450 nm)
Time (days)
shCtrl
shcGAS
ns
N
OCI-LY19
Relative cGAS mRNA levels
shCtrl
shcGAS
O
OCI-LY19
OD value (450 nm)
Time (days)
shCtrl
shcGAS
ns
P
OCI-LY19
OD value (450 nm)
Time (days)
shCtrl
shCtrl + eccDNA+
shSTING
shSTING + eccDNA+

Figure S7

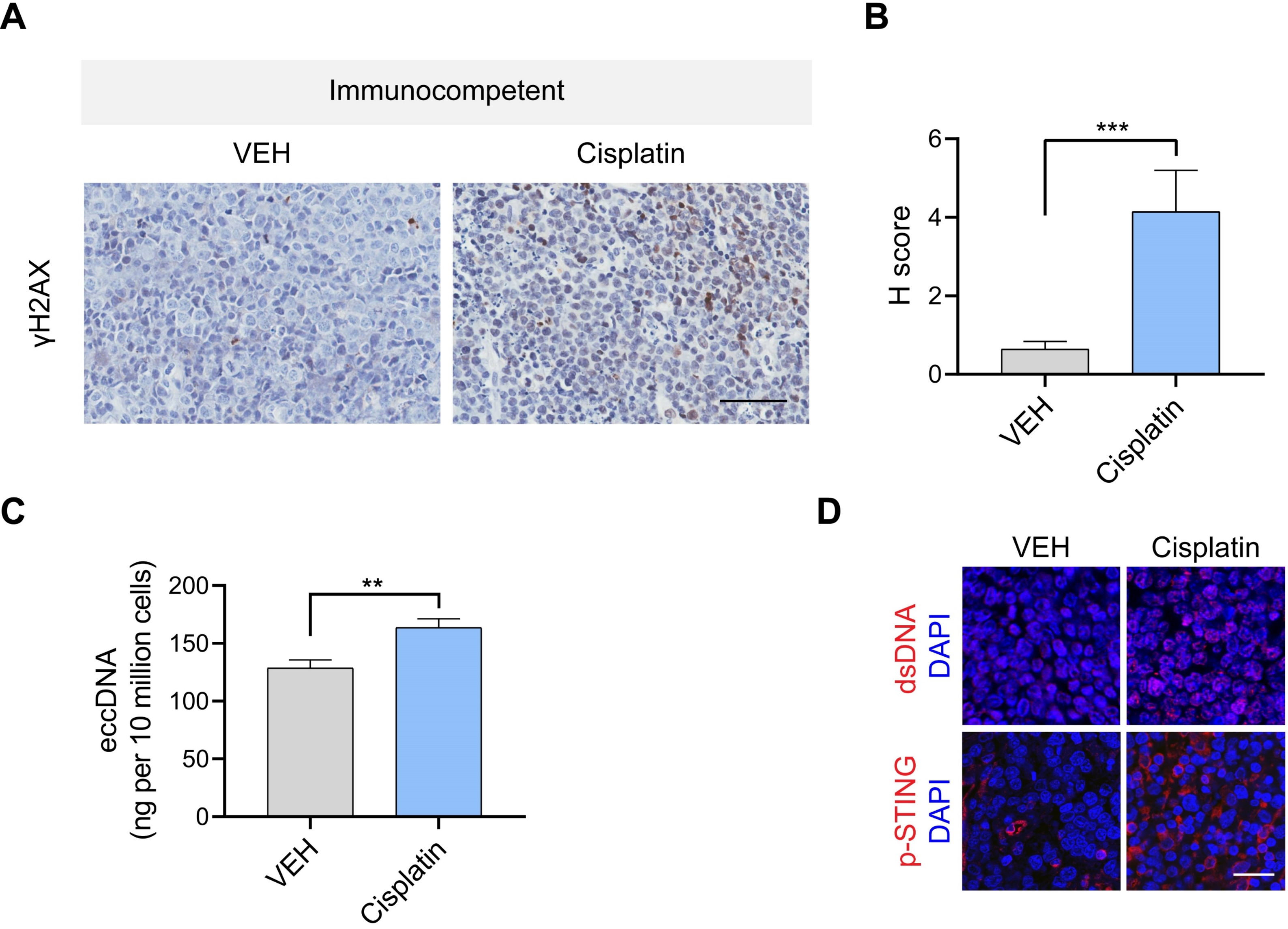
A
Immunocompetent
VEH
Cisplatin
γH2AX
B
***
H score
6
4
2
0
VEH
Cisplatin
C
**
eccDNA
(ng per 10 million cells)
200
150
100
50
0
VEH
Cisplatin
D
VEH
Cisplatin
dsDNA DAPI
p-STING DAPI

Figure S8

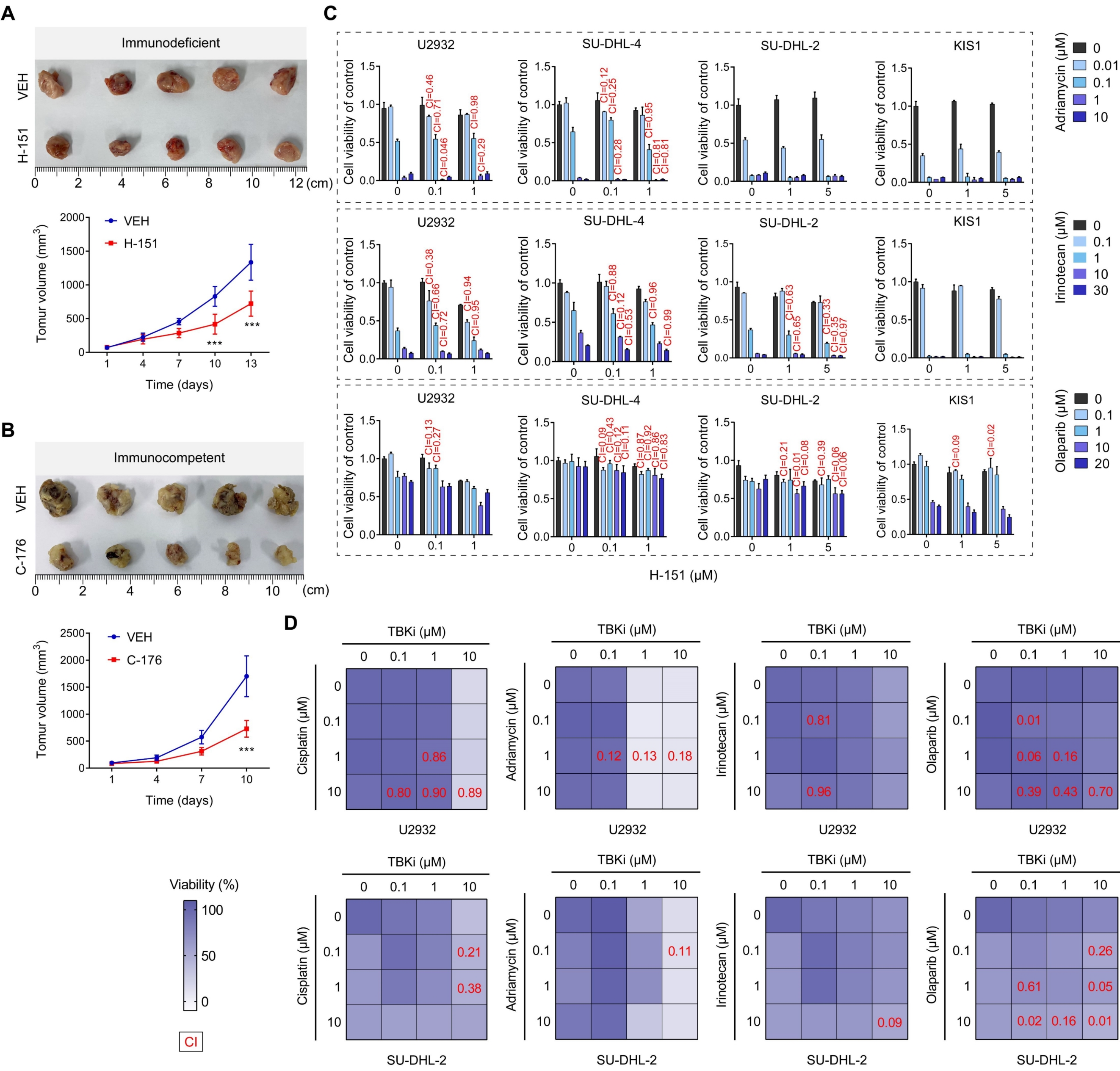
A
Immunodeficient
VEH
H-151
0 2 4 6 8 10 12 (cm)
Tomur volume (mm3)
VEH
H-151
Time (days)
***
B
Immunocompetent
VEH
C-176
0 2 4 6 8 10 (cm)
Tomur volume (mm3)
VEH
C-176
Time (days)
***
C
U2932
SU-DHL-4
SU-DHL-2
KIS1
Cell viability of control
Adriamycin (μM)
0
0.01
0.1
1
10
CI=0.46
CI=0.71
CI=0.046
CI=0.98
CI=0.29
CI=0.12
CI=0.25
CI=0.28
CI=0.95
CI=0.81
CI=0.81
Irinotecan (μM)
0
0.1
1
10
30
CI=0.38
CI=0.66
CI=0.72
CI=0.94
CI=0.95
CI=0.88
CI=0.12
CI=0.53
CI=0.96
CI=0.99
CI=0.63
CI=0.65
CI=0.33
CI=0.35
CI=0.97
Olaparib (μM)
0
0.1
1
10
20
CI=0.13
CI=0.27
CI=0.09
CI=0.43
CI=0.12
CI=0.11
CI=0.87
CI=0.92
CI=0.86
CI=0.83
CI=0.21
CI=0.01
CI=0.08
CI=0.39
CI=0.06
CI=0.06
CI=0.09
CI=0.02
H-151 (μM)
D
TBKi (μM)
Cisplatin (μM)
Adriamycin (μM)
Irinotecan (μM)
Olaparib (μM)
0.86
0.80
0.90
0.89
0.12
0.13
0.18
0.81
0.96
0.01
0.06
0.16
0.39
0.43
0.70
U2932
0.21
0.38
0.11
0.09
0.26
0.61
0.05
0.02
0.16
0.01
SU-DHL-2
Viability (%)
100
50
0
CI

Figure S9

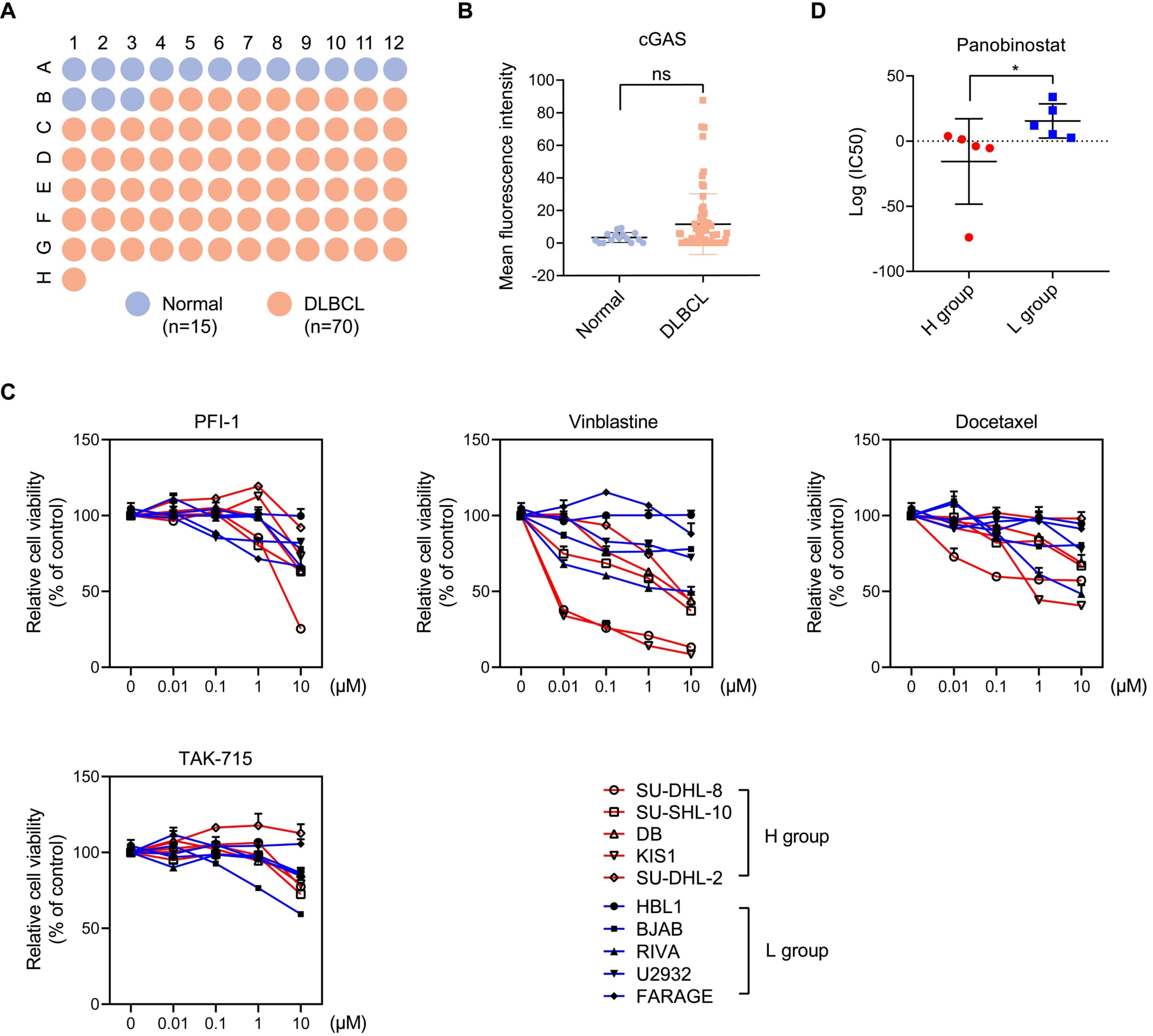

A
1
2
3
4
5
6
7
8
9
10
11
12
A
B
C
D
E
F
G
H
Normal
(n=15)
DLBCL
(n=70)
B
cGAS
ns
Mean fluorescence intensity
Normal
DLBCL
D
Panobinostat
*
Log (IC50)
H group
L group
C
PFI-1
Vinblastine
Docetaxel
TAK-715
Relative cell viability
(% of control)
(μM)
SU-DHL-8
SU-SHL-10
DB
KIS1
SU-DHL-2
H group
HBL1
BJAB
RIVA
U2932
FARAGE
L group
