# Supplementary material for: Multi‐omics integration reveals the oncogenic role of eccDNAs in diffuse large B‐cell lymphoma through STING signalling
Source: Clin Transl Med. 2024 Aug 25;14(8):e1815. doi: 10.1002/ctm2.1815 (PMC11345442; doi:10.1002/ctm2.1815)
Supplement: Supplementary file 2 — Supporting information [file CTM2-14-e1815-s002.docx]

**Supplementary Materials**

**DNA extraction, eccDNA purification, sequencing**

Magnetic Animal Tissue Genomic DNA Kit (TIANGEN, DP341) was used to collect and split cells for obtaining purified HMW (High Molecular Weight) DNAs. The DNA quality was determined using Qubit (Invitrogen). To eliminate linear DNAs and enrich eccDNAs, 10 μg of DNA was digested with Plasmid-Safe ATP-Dependent DNase (20 units) (Lucigen，E3110K) at 37 ℃ for 24 h. DNase and ATP were then added to digest for at least 5 d until digestion was completed. The efficiency and quality of eccDNA enrichment were determined by qPCR with primers targeting COX5B coding genes. The target band cannot be amplified after linear DNAs digestion. According to the provider’s instructions, phi29 polymerase (VAZYME, N106) and random hexamer oligomers were used to amplify eccDNA-enriched DNA samples. Then, the products were fragmented to an average size of 300-500 base pairs using a focused ultrasonicator (Bioruptor™，Diagenode). MolPure® Gel Extraction Kit (YEASEN, 19101) was used for purification. The fragmented DNA samples were used for sequencing library construction (VAHTS Universal DNA Library Prep Kit for Illumina V3, VAZYME, ND607). Next-generation sequencing was conducted using Illumina NovaSeq 6000 using the constructed libraries after the library was purified by using purification beads and the size distribution of fragments was analyzed by Agilent 2100 Bioanalyzer. 2 x 150-bp paired-end reads were sequenced for all libraries.

**Sequencing data analysis and identification of eccDNAs by Circle-MAP**

Before reading the mapping, cutadapt software (v3.5) was used to identify and remove the adapters and any faulty sequences. Then, the clean reads were aligned to the human reference genome (hg38; <http://hgdownload.soe.ucsc.edu/goldenPath/hg38/bigZips/hg38.fa.gz>) using bwa-mem2 software (v 2.2.1). The python package Circle Map (v 1.1.4) was used to identify eccDNAs in all samples. The annotations of eccDNAs were generated using Bedtools (v 3.5.1) with human genomic features (hg38;ftp://ftp.ebi.ac.uk/pub/databases/gencode/Gencode_human/release_38/gencode.v38.annotation.gtf.gz). Genomic coverage was calculated using bamCoverage from deeptools (v 3.5.1) with binSize 1.

**General characteristics of eccDNAs**

After locating the eccDNA molecules in the reference genome, we evaluated their general characteristics, including the length distribution, GC content, and preference for chromosome origin. All plots were generated using the R package ggplot2 (v 3.3.5). The standardized chromosome origin was obtained by dividing the count of eccDNAs by the chromosome size in Mb. R package RIdeogram (v 0.2.2) was used to visualize the chromosome origin of eccDNAs.

**Analysis of the differentially expressed eccDNAs**

To find differentially expressed eccDNAs, the R package edgeR (v 3.32.1) was used, and P < 0.05 was used as a cut-off value.

**ScRNA-seq, primary analysis of raw read data**

Single-cell suspensions (2×10^5^ cells/mL) resuspended in phosphate buffered saline (PBS) were loaded onto the microwell chip using the Singleron Matrix® Single Cell Processing System. ScRNA-seq libraries were constructed using the GEXSCOPE® Single Cell RNA Library Kits (Singleron) ^[1]^. Raw reads from scRNA-seq were processed to generate gene expression matrixes using the CeleScope (https://github.com/singleron-RD/CeleScope) v 1.9.0 pipeline. Briefly, raw reads were first processed with CeleScope to remove low quality reads with Cutadapt v1.17 to trim poly-A tail and adapter sequences. Cell barcode and UMI were extracted. After that, we used STAR v2.6.1a to map reads to the reference genome GRCh38 (ensembl version 92 annotation). UMI counts and gene counts of each cell were acquired with featureCounts v2.0.1software, and used to generate expression matrix files for subsequent analysis.

**scRNA-seq based CNA detection**

The InferCNV package was used to detect the CNAs in DLBCL cells. Normal B cells from healthy volunteers were used as baselines to estimate the CNAs of malignant cells. Genes expressed in more than 20 cells were sorted based on their loci on each chromosome. The relative expression values were centered to 1, using 1.5 standard deviation from the residual-normalized expression values as the ceiling. A slide window size of 101 genes was used to smoothen the relative expression on each chromosome, to remove the effect of gene-specific expression. The CNV score of each cell was calculated as quadratic sum of CAN region.

**ShRNAs and transfection**

Targeted short hairpin RNAs (shRNAs) (Supplementary Table S2) were designed and synthesized by GeneChem Biomedical Co. Ltd. (Shanghai, China). Lentivirus was transfected into DLBCL cells at an optimal multiplicity of infection (MOI) of 100, supplemented with enhanced infection solution and polybrene, according to the manufacturer’s protocol. For eccDNA transfection, 2 million DLBCL cells were plated in a 24-well plate and cultured in OPTI-MEMI medium (Gibco). Cells were then transfected with the eccDNA-Hieff Trans complex, according to provider’s instructions, using Hieff Trans (Yeasen, Shanghai, China).

**Western blotting**

Western blotting was performed using methods as previously described ^[2]^. Antibodies used are listed in Supplementary Table S2.

**RNA extraction and quantitative real-time PCR**

Total RNAs isolated from cells were reverse-transcribed to cDNA using HiScript® III RT SuperMix for qPCR (Vazyme). Quantitative Real-Time PCR (qRT-PCR) was performed by using Taq Pro Universal SYBR qPCR Master Mix (Vazyme) with specific primers listed in Supplementary Table S2.

**Flow cytometry**

For apoptotic analysis, cells were washed twice with PBS and collected. 500 μl Binding Buffer was added to suspend the cells, and then 5 μl of Annexin V-FITC and 5 μl of Propidium Iodide (APExBIO, Houston, USA) were added and mixed well. The reaction was conducted in dark at room temperature for 15 minutes, and the cells were analyzed on a BD FACS LSR II flow cytometer (BD Biosciences, Heidelberg, Germany).

**Immunohistochemistry (IHC) staining**

The paraffin-embedded sections were cut at a 4 thickness of mm, deparaffinized, and rehydrated. Antigen retrieval was performed, and then samples were blocked with normal goat serum. Subsequently, they were probed overnight with primary antibodies. After being washed with PBS, the slides were incubated with a secondary antibody (1:100 in PBS, 1% BSA, and 1% serum) for 30 minutes. Staining was visualized using the 3, 3′-diaminobenzidine tetrahydrochloride (Sigma) for 10 minutes, followed by counterstaining with haematoxylin for 2 min. Finally, the slides were covered with mounting medium. Antibodies used were listed in Supplementary Table S2.

**Immunofluorescence staining**

Treated cells were co-stained overnight at 4°C with primary antibodies for dsDNA and p-STING (Cell Signaling Technology). FITC-conjugated goat anti-rabbit antibody (Abcam) was then applied as a secondary antibody. Nuclei were stained with DAPI (Abcam). Images were acquired using a fluorescence microscope from Leica, Germany. Antibodies used were listed in Supplementary Table S2.

**Chromosome karyotype analysis**

Cells were stimulated with DSP30 and IL-2, and cultured at 37 ° C for 72 h for karyotype analysis. Colchicine (10 mg/L) was added 3 h before culture termination to achieve a final mass concentration of 0.5 μg/ml. After centrifugation and discarding the supernatant, cells were treated with 0.075 mol/ml potassium chloride, and then fixed three times with a 3:1 methanol and ice ethanol solution. Finally, karyotype analysis was carried out using the Giemsa reverse banding technique.

**Comet assay**

Commet assay was performed with the DNA Damage Detection Kit (#KGA240-100, KeyGene Biotech, Nanjing, China) following the provided protocol. Briefly, treated cells were collected and washed with cold PBS. Three layers of gel were prepared and added to a glass slide, which was then lysed by lysis buffer at 4 ℃ for 1 to 2 h. The slide was placed in the horizontal electrophoresis tank, and alkaline electrophoresis buffer (1 mmol/LEDTA, 300 mmol/L NaOH) was poured onto the gel surface. The slide was left at room temperature for 20-60 min. After DNA alkaline unwinding, the slide was electrophoresed for 20-30 min. Following neutralization and dying, the nuclear DNA and migrated DNA can be clearly observed with fluorescence microscope.

**Cell counting kit 8 (CCK8)**

For cell proliferation analysis, 1×10^5^ DLBCL cells were plated in 96-well plates (Thermo, USA) with 0.1 mL culture medium per well. 10 μL CCK8 solution (APExBIO) was added to each well at pointed time. After incubation for 3 h, the absorbance at 450 nm was measured using a microplate reader (Multiskan FC). The experiment was conducted three times. The drugs used in this study (Supplementary Table S2) were dissolved in dimethyl sulfoxide (DMSO) and stored at -20 °C until use. The combination indexes (CIs) analyzed by Chou Talalay algorithm were used to determine the synergistic effects of the drugs. Synergistic, additive, and antagonistic effects were defined as CI < 1, CI = 1, and CI > 1, respectively.

**Atomic force microscope (AFM)**

AFM imaging of eccDNAs was conducted according to the reported protocol ^[3]^. Images were acquired with the Dimension Icon.

**Supplementary Figures**

**Fig. S1. Characterizations of eccDNAs in 18 DLBCL cells lines.**

(A) The correlation between the number and abundance of eccDNAs in 18 DLBCL cell lines. (B) The correlation between the weighted genome instability index (wGII) and eccDNA abundance. (C) The size distribution of eccDNAs in cells from GCB and non-GCB groups. (D) Correlation between the results of eccDNA abundance detected by AGE and circle-seq in different DLBCL cell lines. (E) Number of eccDNAs in GCB and non-GCB DLBCL groups. (F) Heatmap shows the differentially expressed eccDNAs in H and L groups. (G) GC contents of eccDNA locus and regions immediately upstream and downstream from the eccDNAs, compared to the genomic average in GCB and non-GCB DLBCL cell lines. upstream, upper panel; downstream, lower panel; Orange, 1000 stretches upstream eccDNA locus (from eccDNA_start-1000 to eccDNA_start); Red, eccDNA (from eccDNA_start to eccDNA_end); Green, 1000 stretches downstream eccDNA locus (from eccDNA_end to eccDNA_end+1000); Purple, 1000 random stretches of the genome of equivalent length as the eccDNA. (H) Normalized mapping ratio of eccDNA reads in different types of elements from GCB and non-GCB groups.

**Fig. S2. Chromosome karyotypes of 11 GCB DLBCL cell lines.** Red: H group; Blue: L group.

**Fig. S3. Chromosome karyotypes of 7 non-GCB DLBCL cell lines.** Red: H group; Blue: L group.

**Fig. S4. The identified TFs in 18 DLBCL cell lines (A), and in H and L groups (B) based on scRNA-seq data.** Red: H group; Blue: L group.

**Fig. S5. The relationship between eccDNA abundance and genes related to proliferation.**

(A) Correlation between the levels of MAD21, DUT, and eccDNA abundance in DLBCL cell lines. (B) ROC curve of NOP56 is built to differentiate H and L groups.

**Fig. S6. EccDNAs promote cell proliferation through STING pathway in a cGAS-independent manner.**

(A) EccDNAs extracted from the KIS1 cell line in H group are transfected into the OCI-LY19 cell line in L group. (B) The quantified abundance of eccDNAs in the eccDNA+ (cells with eccDNA transfection) and ctrl groups (cells without eccDNA transfection). (C) Analysis of the proliferative ability of cells from eccDNA+ and ctrl groups. (D) Survival analysis of genes that highly expressed in eccDNA+ group from independent DLBCL cohorts. (E) Unsupervised clustering of cells from ctrl and eccDNA+ groups by t-SNE plot. (F) The proportion of each cluster in ctrl and eccDNA+ groups. The dashed lines represent 80 % and 20 %, respectively. (G) GSVA shows the top 10 enriched pathways in different clusters. (H) The expression of proteins in the cGAS-STING pathway in OCI-LY19 cells with or without eccDNA transfection. Representative images of three independent experiments. (I) IF staining shows the levels of p-STING in eccDNA+ and ctrl cells. Scale bar, 50 μm. (J) Relative levels of STING after STING knockdown. Representative images of three independent experiments. (K) The growth curve of cells with different levels of STING. (L-O) Knockdown efficiency of cGAS and the growth curve of U2932 and OCI-LY19 cells with different levels of cGAS. (P) Growth curves of OCI-LY19 cells with cGAS silencing and/or eccDNA transfection. P values were calculated by two-tailed Student’s t-test (B, J, L, N), two-way ANOVA (C, K, M, O, P), log-rank test (D). ns, not significant; *p < 0.05, **p < 0.01, ***p < 0.001.

**Fig. S7. DNA-damaging chemotherapeutic agents promote the generation of eccDNAs and activation of STING in** **immunocompetent mice.**

(A-D) Representative IHC staining for γH2AX, scale bar, 50 μm, (A), IHC score of γH2AX (B), eccDNA abundance (C), and IF staining for dsDNA and p-STING (D) in tumors from immunocompetent mice treated with vehicle or cisplatin. Scale bar, 20 μm. All results are expressed as the mean ± SD of three independent experiments. P values were calculated by two-tailed Student’s t-test. *p < 0.05, **p < 0.01, ***p < 0.001.

**Fig. S8. Chemotherapeutic drugs exert synergistic anti-tumor effects with the inhibitors of STING and TBK1.**

(A, B) The anti-tumor effects of STING inhibitors H-151 and C-176 in immunocompetent (A) and immunodeficient (B) mouse models respectively. (C) CCK8 analysis of proliferation activity in U2932, SU-DHL-4, SU-DHL-2, and KIS1 cell lines treated with H-151 combined with DNA-damaging agents. (D) The synergistic effects of TBK1 inhibitor and DNA-damaging agents. All results are expressed as the mean ± SD of three independent experiments. P values were calculated by two-way ANOVA (A, B). ***p < 0.001.

**Fig. S9. The cGAS/STING expression exhibits heterogeneity and eccDNA abundance is associated with drug sensitivity.**

(A) A tissue chip composed of 70 DLBCL tumor tissues and 15 normal lymph nodes. (B) IF staining shows the levels of cGAS between normal and DLBCL patients. ns, not significant. (C) CCK8 detects the proliferation ability of five cell lines from H group and five cell lines from L group with the treatment of PFI-1, vincristine, docetaxel, TAK-715, and vinblastine. (D) Comparing the IC50 values of panobinostat between H and L groups. All results are expressed as the mean ± SD of three independent experiments. P values were calculated by two-tailed Student’s t-test (B, D). ns, not significant; *p < 0.05.

**Supplementary Tables**

**Table S1. Characteristics of 18 DLBCL cell lines.**

| **Cell line** | **GCB/non-GCB group** | **Karyotype** | **H/L group** | **Original eccDNA numbers** | **Filter eccDNA numbers** | **Total length of eccDNA** | **Media length of eddDNA** | **Abundance of eccDNA** |
| --- | --- | --- | --- | --- | --- | --- | --- | --- |
| BJAB | GCB | 45-47,XX,del(2)(q32q33),del(3)(p14),add(4)(q25),+5,del(6)(p21),add(8)(q24),add(10)(p12),del(11)(q23),-13,add(14)(q32),-15,+16,der(17)t(7;17)(p15;p11)del(17)(q25),-22[20] | L | 3100 | 1712 | 22404564 | 486 | 0.05278899 |
| CTB-1 | GCB | 48,XY,+1,del(1)(p11p35),t(3;12)(q27;p13),t(6;20)(p10;q10),+7,t(8;14)(q24;q32),del(11)(q21),+12,t(14;22)(q32;q11),-16[20] | L | 130834 | 99222 | 3608081414 | 407 | 1.06090763 |
| DB | GCB | 71-76,XXY,+Y,-2,del(3)(p14),add(4)(p16),del(5)(q22),+6,del(6)(p21)x2,+8,del(8)(q24),der(8)add(8)(p11)del(8)(q24),+11,del(11)(q21),+13,add(13)(q34),del(13)(q12q22)x2,t(14;18)(q32;q21),-15,+16,del(16)(p13),del(17)(p13),+der(18)t(14;18),+19,+20[20] | H | 6359 | 4088 | 592610291 | 423 | 0.20964818 |
| DOHH2 | GCB | 47,XY,+7,t(8;14)(q24;q32),t(14;18)(q32;q21)[20] | L | 324332 | 270074 | 8335877184 | 378 | 1.09229617 |
| FARAGE | GCB | 46,XX,del(6)(p21)[20] | L | 9207 | 6863 | 380159547 | 429 | 0.0449547 |
| HBL1 | non-GCB | 87-89,XXX,del(X)(q24),+del(2)(q32q33),del(4)(p31)x2,+5,add(6)(p22)x2,-7,add(11)(q25)x2,add(12)(p13),der(13;16)(q10;q10)x2,-17,add(17)(p11),-18,add(19)(q13)x2[20] | L | 1063 | 850 | 519191 | 366 | 0.13569173 |
| KIS1 | non-GCB | 45,X,add(X)(q28),del(1)(q12),t(3;19)(q27;q13),del(6)(q21),del(7)(q11),der(8)del(8)(p11)t(8;22)(q24;q11),-9,der(9)t(9;14)(p13;q12),add(10)(q26),add(12)(p13),add(13)(q31),add(14)(q32),der(18)t(14;18)(q32;q21)[20] | H | 1666 | 983 | 12819416 | 734 | 0.04596493 |
| MEDB1 | non-GCB | 48-49,XY,+1,der(1)del(1)(p35)del(1)(q25),+2,del(2)(q23q32),add(6)(q27),+8,+9,der(12;14)(q10;q10),add(21)(p11)[20] | L | 5678 | 3809 | 560640984 | 367 | 0.3128168 |
| OCI-LY19 | GCB | 48,XX,+6,del(6)(q15)x2,+8,del(8)(q24),t(14;18)[20] | L | 11493 | 8530 | 130248648 | 470 | 0.34486346 |
| OCI-LY3 | non-GCB | 68-73,XXY,+Y,+1,der(1)t(1;17)(p13;q12)x2,+3,-4,del(6)(q13)x2,+9,-10,+12,-15,-17,+19,add(19)(q13)x2,+20,+22[20] | H | 4216 | 3069 | 531680181 | 422 | 0.0877869 |
| RIVA | non-GCB | 86-94,XXX,add(X)(q26),del(1)(p34)x2,del(3)(q10)x2,der(3)t(3;3)(q27;q27)x2,t(4;8)(q21;q24)x2,del(6)(q10)x2,+del(9)(q32),add(14)(q32)x2,der(15)t(13;15)(q13;p11)x2,add(17)(q25)x2,del(18)(q21)x2,+mar[20] | L | 4369 | 3757 | 29040367 | 237 | 0.08360006 |
| SU-DHL-2 | non-GCB | 49-53,XX,del(2)(q31),+del(3)(p11),t(3;22)(q27;q11),+4,+5,del(6)(q21q23),+7,der(7)t(7;13)(p22;q14),+9,del(9)(q32),+11,+15,+20,del(20)(q11)[20] | H | 175335 | 143238 | 5384242513 | 378 | 0.94194276 |
| SU-DHL-4 | GCB | 48-50,XY,+X,-3,dup(3)(q22q24),+7,+12,der(12),+13,del(13)(q14q21),t(14;18)(q32;q21),add(16)(p13),+18,der(18)t(14;18)[20] | L | 85836 | 58884 | 807359009 | 553 | 0.89889842 |
| SU-DHL-6 | GCB | 46-49,XY,del(4)(q21),+8,der(8)t(8;8)(p23;q21),del(8q24),del(9)(p13),del(11)(q21),t(14;18)(q32;q21),+16,t(16;22)(q10;q10),+der(18)t(14;18)[20] | H | 700 | 576 | 879619 | 804.5 | 0.09473698 |
| SU-DHL-8 | GCB | 51,X,-Y,+6,add(6)(p21),+7,t(8;22)(q24;q11),+13,+16,der(16)t(3;16)(p12;p10),+20,+20,del(20)(q13)[20] | H | 243469 | 208935 | 4013540177 | 381 | 1.09839113 |
| SU-DHL-10 | GCB | 44-47,XY,+7,der(8)add(8)(p23)del(8)(q24),del(10)(q22q24),der(11)t(Y;11)(q11;q25),der(14)t(8;14)(q24;q32),der(18)t(14;18)(q32;q21)[20] | H | 3487 | 2180 | 2624243 | 422.5 | 0.05378936 |
| U2932 | non-GCB | 45-46,X,add(X)(q22),del(1)(q21)x2,t(3;18)(q11;q21),add(4)(q21),add(5)(q32)x2,del(6)(q15),del(10)(q23),t(8;14)(q24;q32),-13,add(13)(q32),-14,del(15)(q15),+18,add(18)(q22),del(19)(q13)[20] | L | 49872 | 35850 | 1202028169 | 527 | 0.83683951 |
| WSU-DLCL2 | GCB | 46-48,XY,t(1;2)(p36;q37),del(3)(q25),t(4;14)(q27;q31),i(7)(p10),+8,+der(11)add(11)(p11)del(11)(q23),del(13)(q21q22),t(14;18)(q32;q21),add(15)(p11),del(16)(p11)[20] | L | 2592 | 2100 | 25180474 | 381 | 0.12492036 |

**Table S2. Reagents and resource used in this article.**

| **REAGENT or RESOURCE** | **SOURCE** | **IDENTIFIER** |
| --- | --- | --- |
| **Oligonucleotides** |  |  |
| CENPK Forward: 5′-ACCGCTGAACTCAGTCAATGGC-3′ | This paper | NA |
| CENPK Reverse: 5′-TTGACTCCTTAGTGGACAGTACC-3′ | This paper | NA |
| cGAS Forward: 5′-AGGAAGCAACTACGACTAAAGCC-3′ | This paper | NA |
| cGAS Reverse: 5′-CGATGTGAGAGAAGGATAGCCG-3′ | This paper | NA |
| DEK Forward: 5′-TGGGTCAGTTCAGTGGCTTTCC-3′ | This paper | NA |
| DEK Reverse: 5′-CTCTCCAAATCAAGAACCTCACAG-3′ | This paper | NA |
| DUT Forward: 5′-CCTTCTGGGTGTTATGGAAGAGT -3′ | This paper | NA |
| DUT Reverse: 5′-GCTGTGCAATTCGATCACCTTT-3′ | This paper | NA |
| GAPDH Forward: 5′-CTGGGCTACACTGAGCACC-3′ | This paper | NA |
| GAPDH Reverse: 5′-AAGTGGTCGTTGAGGGCAATG-3′ | This paper | NA |
| H2AFZ Forward: 5′-GCAACTTGCTATTCGTGGAGATG-3′ | This paper | NA |
| H2AFZ Forward: 5′-GCAACTTGCTATTCGTGGAGATG-3′ | This paper | NA |
| H2AFZ Reverse: 5′-CAGGCATCCTTTAGACAGTCTTC-3′ | This paper | NA |
| H2AFZ Reverse: 5′-CAGGCATCCTTTAGACAGTCTTC-3′ | This paper | NA |
| HIST1H4C Forward: 5′-CGGGATAACATCCAGGGCATTAC-3′ | This paper | NA |
| HIST1H4C Reverse: 5′-GTATAGGTGACGGCGTCTCGAA-3′ | This paper | NA |
| IFI27 Forward: 5′-CGTCCTCCATAGCAGCCAAGAT-3′ | This paper | NA |
| IFI27 Reverse: 5′-ACCCAATGGAGCCCAGGATGAA-3′ | This paper | NA |
| IFITM2 Forward: 5′-GGCTTCATAGCATTCGCGTACTC-3′ | This paper | NA |
| IFITM2 Reverse: 5′-AGATGTTCAGGCACTTGGCGGT-3′ | This paper | NA |
| IFNA2 Forward: 5′-TGGGCTGTGATCTGCCTCAAAC-3′ | This paper | NA |
| IFNA2 Reverse: 5′-CAGCCTTTTGGAACTGGTTGCC-3′ | This paper | NA |
| ISG15 Forward: 5′-CTCTGAGCATCCTGGTGAGGAA-3′ | This paper | NA |
| ISG15 Reverse: 5′-AAGGTCAGCCAGAACAGGTCGT-3′ | This paper | NA |
| MAD2L1 Forward: 5′-TTGAGTGTGACAAGACTGCAAAAG-3′ | This paper | NA |
| MAD2L1 Reverse: 5′-CAGTGGCAGAAATGTCACCGTAG-3′ | This paper | NA |
| MX1 Forward: 5′-GGCTGTTTACCAGACTCCGACA-3′ | This paper | NA |
| MX1 Reverse: 5′-CACAAAGCCTGGCAGCTCTCTA-3′ | This paper | NA |
| MYBL2 Forward: 5′-CCTTTTTGCCACTTGGGAGTTGG-3′ | This paper | NA |
| MYBL2 Forward: 5′-CTCAGGTCACACCAAGCATCAG-3′ | This paper | NA |
| MYBL2 Reverse: 5′-CACCAGAAACGAGCCTGCCTTA-3′ | This paper | NA |
| MYBL2 Reverse: 5′-GTGCTTGGTTCTTCCACCTCTG-3′ | This paper | NA |
| NOP56 Forward: 5′-GGCTAAGGCTATTCTGGATGCC-3′ | This paper | NA |
| NOP56 Reverse: 5′-TGTGTAGGCTCTGGCGGTATTC-3′ | This paper | NA |
| PCLAF Forward: 5′-CCTTTTTGCCACTTGGGAGTTGG-3′ | This paper | NA |
| PCLAF Reverse: 5′-GTGCTTGGTTCTTCCACCTCTG-3′ | This paper | NA |
| PCNA Forward: 5′-CAAGTAATGTCGATAAAGAGGAGG-3′ | This paper | NA |
| PCNA Reverse: 5′-GTGTCACCGTTGAAGAGAGTGG-3′ | This paper | NA |
| PRDX4 Forward: 5′-CGCTTTTGGCGACAGACTTGAAG-3′ | This paper | NA |
| PRDX4 Reverse: 5′-CCAAGTCCTCCTTGTCTTCGAG-3′ | This paper | NA |
| PSME1 Forward: 5′-TGATGACCAGCCTCCACACCAA-3′ | This paper | NA |
| PSME1 Reverse: 5′-TACTCTGCCTCATCCAGCTCGT-3′ | This paper | NA |
| RANBP1 Forward: 5′-ACCATGACCCTCAGTTTGAGCC-3′ | This paper | NA |
| RANBP1 Reverse: 5′-AGTGCCTCGCTCCTTCCATTCT-3′ | This paper | NA |
| SRSF2 Forward: 5′-CCCGATGTGGAGGGTATGAC-3′ | This paper | NA |
| SRSF2 Reverse: 5′-GAGACTTCGAGCGGCTGTAG-3′ | This paper | NA |
| STING Forward: 5′-CCTGAGTCTCAGAACAACTGCC-3′ | This paper | NA |
| STING Reverse: 5′-GGTCTTCAAGCTGCCCACAGTA-3′ | This paper | NA |
| CGAS-RNAi forward: 5’-GCCTTCTTTCACGTATGTACC-3’ | GENECHEM | NA |
| STING1-RNAi forward: 5’-GCCCGGATTCGAACTTACAAT -3’ | GENECHEM | NA |
| **Antibodies** |  |  |
| Anti-mouse IgG, HRP-linked Antibody | Cell Signaling | Cat# 7076;RRID: AB_330924 |
| Anti-rabbit IgG, HRP-linked Antibody | Cell Signaling | Cat# 7074;RRID: AB_2099233 |
| beta Actin Antibody | Abcam | Cat# ab70165;RRID: AB_1209525 |
| Caspase3/p17/p19 Polyclonal antibody | Proteintech | Cat# 19677-1-AP;RRID: AB_10733244 |
| cGAS (E5V3W) Rabbit mAb | Cell Signaling | Cat# 79978;RRID: AB_2905508 |
| c-Myc (E5Q6W) Rabbit mAb | Cell Signaling | Cat# 18583;RRID: AB_2895543 |
| GAPDH (D16H11) XP® Rabbit mAb | Cell Signaling | Cat# 5174; RRID: AB_10622025 |
| IRF-3 (D6I4C) XP® Rabbit mAb | Cell Signaling | Cat# 4302;RRID: AB_1904036 |
| Mouse Anti-double Stranded DNA Monoclonal Antibody | Millipore | Cat# MAB10246;RRID: AB_1977511 |
| PARP1 Polyclonal antibody | Proteintech | Cat#13371-1-AP; |
| PCNA (D3H8P) XP® Rabbit mAb | Cell Signaling | Cat# 13110;RRID: AB_2636979 |
| Phospho-CGAS-Y215 Rabbit pAb | ABclonal | Cat# RK05796;RRID: AB_2864228 |
| Phospho-Histone H2A.X (Ser139) (20E3) Rabbit mAb | Cell Signaling | Cat# 4309;RRID: AB_10694395 |
| Phospho-IRF-3 (Ser386) (E7J8G) XP® Rabbit mAb | Cell Signaling | Cat# 4947;RRID: AB_823547 |
| Phospho-STING/TMEM173-S366 Rabbit pAb | ABclonal | Cat# AP1199;RRID: AB_2864054 |
| Phospho-TBK1/NAK (Ser172) (D52C2) XP® Rabbit mAb | Cell Signaling | Cat# 5483;RRID: AB_10693472 |
| TBK1/NAK (D1B4) Rabbit mAb | Cell Signaling | Cat# 3504;RRID: AB_2255663 |
| TMEM173/STING Polyclonal antibody | Proteintech | Cat#19851-1-AP;RRID: AB_10665370 |
| **Chemicals** |  |  |
| C171 | Selleck | Cat# E0128 |
| C176 | Selleck | Cat# S6575 |
| Cisplatin | Selleck | Cat# S1166 |
| DiABZI STING agonist | Selleck | Cat# S8796 |
| Docetaxel | MedChemExpress | Cat# HY-B0011 |
| Doxorubicin | Selleck | Cat# E2516 |
| G150 | MedChemExpress | Cat# HY-128583 |
| H151 | MedChemExpress | Cat# HY-112693 |
| Irinotecan | Selleck | Cat# S1198 |
| Olaparib | MedChemExpress | Cat# HY-10162 |
| Panobinostat | MedChemExpress | Cat# HY-10224 |
| PCNA-I1 | Selleck | Cat# S4476 |
| PFI-1 | MedChemExpress | Cat# HY-16586 |
| TAK-715 | MedChemExpress | Cat# HY-10456 |
| TBK1/IKKε-IN-1 | Selleck | Cat# S8922 |
| Vincristine | MedChemExpress | Cat# HY-N0488A |

**References**

1. Dura B, Choi JY, Zhang K, Damsky W, Thakral D, Bosenberg M*, et al.* scFTD-seq: freeze-thaw lysis based, portable approach toward highly distributed single-cell 3' mRNA profiling. **Nucleic acids research** **2019**, 47(3)**:** e16.

2. Wu Z, Wang L, Fan L, Tang H, Zuo X, Gu D*, et al.* Exploring the significance of PAK1 through chromosome conformation signatures in ibrutinib-resistant chronic lymphocytic leukaemia. **Mol Oncol** **2022**, 16(16)**:** 2920-2935.

3. Wang Y, Wang M, Djekidel MN, Chen H, Liu D, Alt FW*, et al.* eccDNAs are apoptotic products with high innate immunostimulatory activity. **Nature** **2021**, 599(7884)**:** 308-314.
